# New climate regime restructures the ecology of Canada’s Northern Great Lakes

**DOI:** 10.1073/pnas.2603271123

**Published:** 2026-06-01

**Authors:** Kathleen M. Rühland, Neal Michelutti, Marlene S. Evans, Kimberly L. Howland, John P. Smol

**Affiliations:** ^a^ Department of Biology, Paleoecological Environmental Assessment and Research Lab, Queen’s University, Kingston, ON K7L 3N6, Canada; ^b^ Environment and Climate Change Canada, Saskatoon, SK S7N 3H5, Canada; ^c^ Arctic Fisheries and Marine Mammal Science Division, Freshwater Institute, Fisheries and Oceans Canada, Winnipeg, MB R3T 2N6, Canada

**Keywords:** Arctic lakes, diatoms, paleolimnology, Great Bear Lake, ice cover

## Abstract

Great Bear Lake (GBL), Great Slave Lake (GSL), and Lake Hazen in northern Canada have recently experienced a shift in fundamental aquatic ecosystem processes associated with accelerated Arctic warming. Multiple high-resolution, ^210^Pb-dated sediment records (GBL: Smith, Keith, McVicar arms, GSL: West Basin, Lake Hazen: Blister, Main sites) registered remarkably similar restructuring of algal community composition over the past few decades in all lakes. A rapid proliferation of small euplanktonic diatoms at the turn of the 21st century displaced benthic (GBL, Lake Hazen) and large filamentous (GSL) taxa that dominated the diatom assemblages since the mid-to-late 1800s. An increase in the number of ice-free days, a rise in regional air temperature, and a decline in wind speed primed the lakes for new physical and thermal regimes that facilitated a shift to greater whole lake primary production and favored small obligate planktonic diatoms. Historical records from surveys undertaken on the lakes between the 1940s and 1970s substantiated our paleolimnological findings that these lakes were relatively stable limnologically since at least the past century and have only recently transitioned to new thermal states. As primary producers fuel the entire food web, this rapid algal community restructuring serves as a cautionary signal of major ecosystem changes. The characteristic thermal inertia and resilience of Canada’s “Northern Great Lakes” is rapidly eroding, with implications for native fish populations that Indigenous and other northern communities rely on.

## Significance

The effects of anthropogenic climate change on large, deep Arctic lakes have been historically buffered by their extensive ice covers. However, recent accelerated warming has now triggered a pronounced response in the algal communities of Canada’s “Northern Great Lakes.” Dated sediment records from Great Bear Lake, Great Slave Lake, and Lake Hazen document an unparalleled and rapid reorganization of primary producers in the past few decades, consistent with 21st century increases in regional air temperature, shorter ice cover periods, and declines in wind speed. Changes in the quantity and composition of algae at the base of the food chain of these large Arctic lakes signal a threshold response that will likely have cascading effects on ecosystem functioning and community structure.

Since the turn of the 21st century, Arctic and sub-Arctic regions have experienced rapid warming (1) in tandem with atmospheric stilling (declines in wind speed) that have had cascading effects on the physical and chemical properties of freshwater systems (2) and ecosystem functioning. Primary producers at the base of aquatic food chains are the essential energy sources that drive the world’s marine and freshwater systems. Diatoms (Bacillariophyceae) are often the dominant primary producers in freshwater lakes and are well-established indicators of climate-mediated change in high latitude regions (3). With progressively longer, warmer, and stiller ice-free periods, essential aquatic ecosystem processes that are inextricably coupled with algal dynamics and species composition are being disrupted (4, 5). For example, increased lake thermal stability (and associated changes in essential light and nutrient resources) has triggered diatom life strategy shifts and increases in algal production in the sediment records of small and medium sized lakes across the circum-Arctic (4–7) including lakes located in Arctic coastal regions where persistent sea ice that once regulated climate is now quickly disappearing (8, 9). In deep and large northern lakes, algal responses are typically delayed as these systems remain ice-covered for much of the year, thereby dampening the full effects of anthropogenic warming (thermal inertia) (3, 10). However, once this critical threshold is surpassed, changes in algal production may be particularly acute in large Arctic lakes where phytoplankton can supply the bulk of energy flow to the rest of the ecosystem (11).

Understanding how global change is impacting the world’s very large and deep high latitude systems is important as they hold a disproportionately large quantity of global surface freshwater (12), play an important role in the global carbon cycle (13, 14), support essential habitats for a large and diverse portion of global species (15), and are typically well-integrated into the social and economic welfare of surrounding populations (14–16). However, large lakes worldwide are becoming increasingly under threat as they are experiencing unprecedented rapid environmental degradation from cumulative and emerging multiple stressors, particularly in heavily populated regions (e.g., Lake Victoria, Africa, and Lake Taihu, China) (17). Although the watersheds of large high latitude lakes typically have relatively low population densities (16) and minimal development (18), they are nonetheless susceptible to the impacts of multiple anthropogenic stressors (18, 19), particularly with accelerated climate change (7, 20–23). For example, colonial bloom-forming cyanobacteria have been generally absent from cold, oligotrophic high-latitude lakes including Great Slave Lake (GSL) (24) but, over the past decade, unprecedented and widespread bloom activity has been reported in sheltered locations of GSL that is likely climate-driven (25).

Despite their unmistakable global significance, basic information on Canada’s large northern lakes such as GBL (Sahtú), GSL (Tu Nedhé, Tucho, Tıdeè, Tinde’e), and Lake Hazen (Tasialuk) is profoundly limited. Over 20 y ago, Schindler (19) wrote that it was “tragic” how little was known about the aquatic communities and biogeochemical processes of GBL, the world’s most pristine great lake, and warned that if human activities continued on its 20th century trajectory, Canada’s “Northern Great Lakes” will clearly be stressed in the 21st century. Since then, scientific research on GBL and GSL has increased (20, 25, 26–33) but nevertheless these iconic sites remain among the most understudied large freshwater systems in the world. Limnological studies are even fewer for Lake Hazen in the extreme High Arctic, but some foundational research was undertaken as early as the late 1950s (34–36) (*SI Appendix*, *Supplementary Text 1*), with more recent work focused on the various impacts of climate warming (37–39). For GBL, community-based fisheries and environmental monitoring programs were established in the early 2000s through collaborative efforts between the Department of Fisheries and Oceans Canada (DFO), the Délınę Renewable Resources Council (DRRC), and the Sahtú Renewable Resources Board (SRRB) (40). For GSL, a community-based monitoring program was initiated by DFO and various Indigenous groups in 2010, and the Government of the Northwest Territories (GNWT) Community-based Water Quality - Water Stewardship Program started in 2012. Although recent research and monitoring programs provide valuable contemporary data, the natural or baseline limnological conditions [as can be inferred from lake sediment archives (paleolimnology)] are required to provide the context for understanding how anthropogenic stressors and recent climate warming are changing the ecosystems of these large northern freshwater lakes.

Here, we assess how accelerated Arctic warming since the end of the 20th century has impacted the ecosystem structure and functioning in three of the largest and deepest freshwater lakes in northern Canada. We examine a series of ^210^Pb-dated lake sediment records collected from three arms of GBL and compare changes in subfossil diatom assemblages over the past ~200 y with recently published diatom records from sub-Arctic GSL (23) to the south, and High Arctic Lake Hazen to the north (21, 22). We compare recent trends in primary production [using visible range spectroscopy-inferred chlorophyll-*a* (VRS-Chla)] from our GBL and GSL sediment records and changes in primary production estimated using satellite remote sensing (14). To independently support (ground-truth) our paleolimnological interpretations, we compile information from historical limnological surveys of GBL (41–46), GSL (24, 47–49), and Lake Hazen (35, 36, 50, 51) including evidence of historic lake mictic state, water surface and water column temperatures, water clarity, pH, nutrients, primary production, and phytoplankton. Trends in our diatom records were compared to available long-term air temperature and wind speed data from nearby weather stations (Norman Wells: GBL, Hay River: GSL, Eureka: Lake Hazen). The Alert climate station was also considered to represent the Lake Hazen region, but climate trends at Eureka best matched available shorter-term instrumental records from Lake Hazen and nearby Tanquary Fiord airport, particularly wind speed (*SI Appendix*, *Supplementary Methods* and Table S3). Last, we compare our paleolimnological trends to available lake ice phenology data including ground observations (GSL), remote sensing data (GSL, GBL) (52), as well as data derived from NASA MODIS daily satellite images (GBL, Lake Hazen).

## GBL (Sahtú).

Below, we specifically provide an overview of GBL properties and historical limnological surveys, given that detailed descriptions of Lake Hazen and GSL have been previously published (21–23). We follow this with a comparison of lake characteristics among the three sites.

GBL (66° 00’N, 120° 56’W), situated within the Sahtú Region of the NWT on the Arctic Circle, is the largest lake [surface area 31,326 km^2^, catchment area (CA) 145,870 km^2^] entirely in Canada, spans three ecozones (Southern Arctic, Taiga Plains, and Taiga Shield), and has a maximum depth of 446.2 m and a mean depth of 71 m (44, 53) (*SI Appendix*, Table S1). It has an irregular shape consisting of five arms [Dease (Tugacho), McTavish (Kwita tla), Smith (Tirato), Keith (Dareli), and McVicar (Turili)] radiating out from a deep central basin, and relative to other comparatively large lakes worldwide, development on its shorelines has been minimal (18). It is considered to be the most pristine example of a truly large, cold, freshwater ecosystem (19, 44, 54, 55) and the largest North American lake that has not been ecologically altered by nonindigenous species introductions (19, 26). Maintaining the ecological integrity of the lake and its watershed is vital to the culture and livelihoods of the Sahtúot’ine of Délınę. In 2016, community leaders successfully petitioned to have GBL designated as a UNESCO Biosphere Reserve (the Tsá Tué Biosphere Reserve), the first in the world located north of 60˚ and the first that is fully managed by Indigenous Peoples.

Historical surveys described GBL as the world’s largest cold-monomictic lake (44, 46) experiencing continuous water column mixing that results in extremely uniform water chemistry and temperatures (42, 44, 46). Recently, Carmack et al. (33) suggested it should now be more accurately described as “hybrid polar” given that it has many characteristics typical of High Arctic (polar) lakes, but some regions of the lake exhibit dimictic characteristics. The lake’s great depth, perennially cold conditions with a short open-water period, together with its relatively small catchment on insoluble bedrock contribute to its ultraoligotrophic nature (45) and its limited biological productivity (42, 44, 46). A 1945 survey of GBL by Miller and Kennedy described its open waters as being representative of a biological desert, concluding that no part of the lake could sustain a large-scale commercial fishery (56). Indeed, a commercial fishery has never been established on GBL. Although it is one of the world’s largest and most recognizable lakes, only a handful of biological and limnological studies have been conducted on GBL since the 1945 inaugural survey. Most notably, Johnson’s 1963 to 1965 surveys (44, 45) provided valuable details on lake physical and chemical characteristics as well as on fish populations, whereas Moore’s 1976 to 1978 nearshore studies reported on algal (46) and zooplankton (57) communities. More recently, the Government of Canada, in collaboration with the DRRC and the SRRB, focused on GBL ecosystem health including changes in climate, water temperature, and fish populations (11, 27). Additionally, mercury and organic contaminants have been monitored in Lake Trout (*Salvelinus namaycush*) since 2008 with GBL selected as a pristine site relative to lakes monitored elsewhere (58).

## Comparison of Lake Characteristics: GBL, GSL, and Lake Hazen.

Globally, GBL, GSL (61° 47’N, 113° 43’W), and Lake Hazen (81° 47’N, 71° 09’W) are considered “large lakes” by the International Association of Great Lakes Research (IAGLR: http://iaglr.org/lakes/) standards (*SI Appendix*, Table S1 and *Supplementary Text 1*). Respectively, GBL and GSL in sub-Arctic (NT) Canada are the 7th and 9th largest freshwater lakes on earth by area and are among the world’s top 10 by lake volume (53). While considerably smaller than GBL and GSL, Lake Hazen (NU) has a greater mean depth (*SI Appendix*, Table S1) and is the largest freshwater lake by volume in the High Arctic (59). Although all three lakes are large and deep, they differ (often substantially) in their degree of remoteness, geographical and landscape setting and other site-specific lake characteristics (*SI Appendix*, Table S1). The southernmost lake (GSL) is also the most densely populated with ~27,260 people living near its shorelines (Statistics Canada, January 2021) and supports the largest commercial, recreational, and Indigenous freshwater fishery in the NWT (31). GBL is approximately 570 km to the north of GSL and has a much smaller population living on its shorelines, with Délınę (population 573, Statistics Canada, 2021 census) the only community on the lake. A well-established sport fishing lodge is located on Dease Arm with Indigenous-subsistence fisheries occurring around the lake (26, 54). In the extreme High Arctic (~2,200 km north of GBL), the truly remote Lake Hazen does not have permanent residents, although it has been used as fishing and hunting grounds for thousands of years by ancient Arctic cultures (60) and seasonally by modern Inuit (39).

Despite its northern continental setting, GBL has many features that are typical of lakes located in the High Arctic islands (e.g., ultraoligotrophic, low biological activity) (44). In contrast, the Lake Hazen region is atypical of its high latitude location as it is protected from prevailing winds by its valley setting and is described as a thermal oasis within a High Arctic polar desert (39). The three lakes differ substantially in their ratios of CA to lake area (LA) with GBL having a remarkably small catchment for such a large lake (CA:LA = 4.7), whereas one of the defining features of GSL is its very large CA [971,000 km^2^ (ref. 44), CA:LA = 33.9] and Lake Hazen is more moderate (CA:LA = 12.7) (*SI Appendix*, Table S1). Lake Hazen and the West Basin of GSL experience relatively short water residence periods [~25 and 7 to 16 y (21, 49), respectively] and high levels of minerogenic turbidity that are in stark contrast with GBL’s very long water residence time (~124 y; ref. 44) and extreme water clarity (*SI Appendix*, Table S1). With accelerated Arctic warming since 2007, Lake Hazen has experienced a dramatic decline in water residence time (from a historical average of ~89 y; ref. 59, to ~25 y; ref. 21), due to a 10-fold increase in the delivery of glacial meltwater and sediment resulting from a rapid loss of mass in catchment glaciers (21, 22). However, ultraoligotrophic conditions have been maintained as meltwater nutrients are bound to particles that are transported below the lake’s photic zone via dense turbidity currents (37). In contrast, the West Basin of GSL receives massive amounts of suspended nutrient- and mineral-rich sediment from its large catchment that is discharged from the Slave River (its main inflow), stimulating primary production that helps support its commercial fishery (24, 48, 49). The three lakes are ice covered for a large portion of the year, with an average ice-free period of approximately 23.5 wk on GSL and 18.5 wk on GBL (61): in recent decades, the once perennially frozen Lake Hazen is now commonly ice-free for a couple of weeks during the summer (21). During the 1940s and 1950s, a well-developed summer thermocline was not observed on GSL and summer thermal stratification was deemed rare (62) and only sporadically observed (24), whereas in GBL the development of persistent summer thermal stratification and a summer thermocline has only been reported within the past decade for most parts of the lake (all arms) (11, 27, 33). Currently, Lake Hazen does not experience summer thermal stratification (37).

## Results and Discussion

**Fig. 1.**
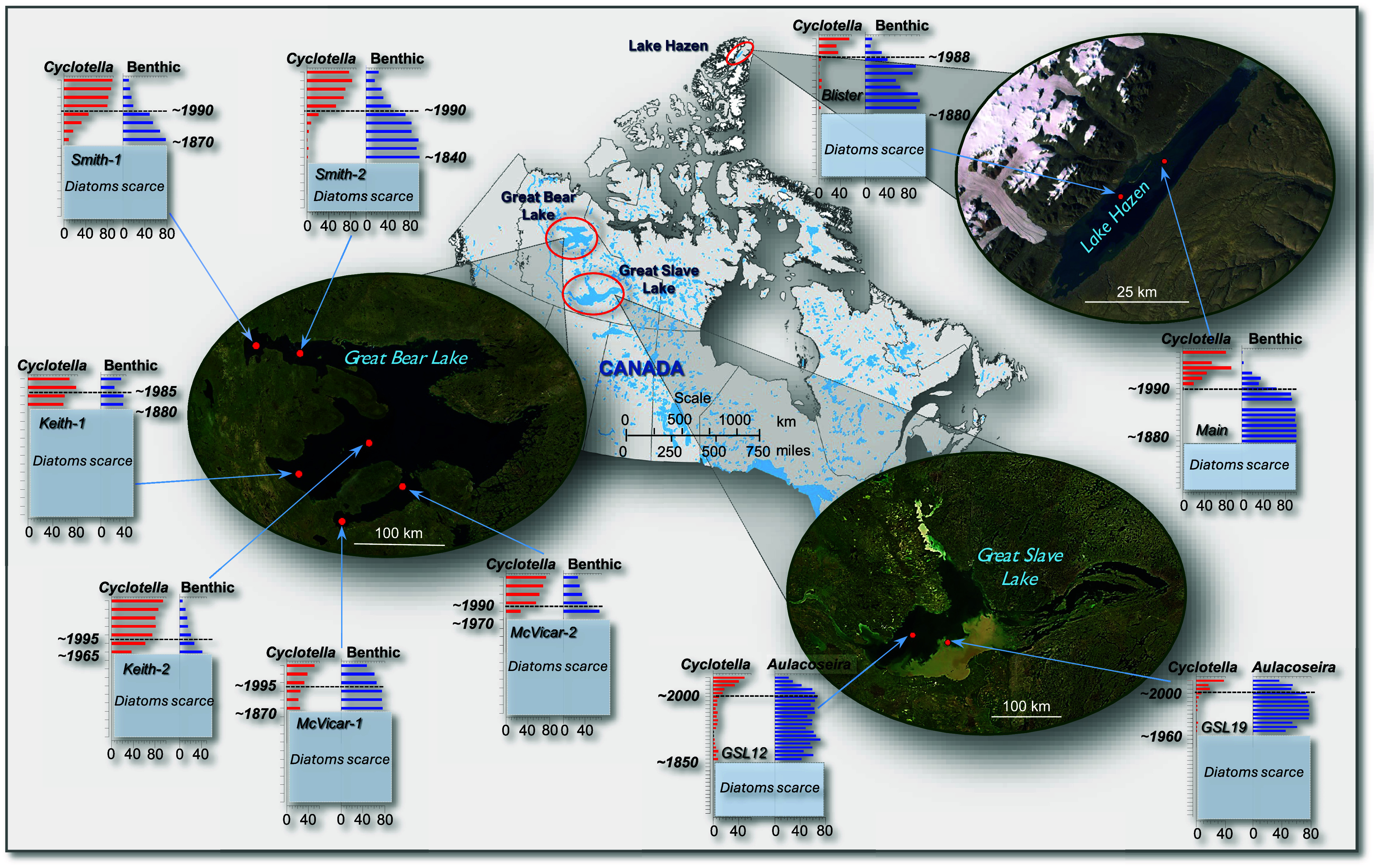
Location of the three “Northern Great Lakes” in Canada and the rise in planktonic cyclotelloid taxa. Diatom compositional changes registered in dated sediment profiles from sub-Arctic [Great Bear Lake (GBL), Great Slave Lake (GSL), NT] and High Arctic (Lake Hazen, NU) Canada, highlighting increases in the percent relative abundance of small, planktonic *Cyclotella* [*sensu lato* (*s*.*l*.)] taxa, since the end of the 20th century. Red dots on satellite images indicate coring locations. The main diatom groups, *Cyclotella* (*s*.*l*.), benthic diatoms, and *Aulacoseira* taxa were each summed in the simplified diatom profiles. GBL: Small *Cyclotella* (s.*l*.) taxa (in order of greatest relative abundance) include *Pantocsekiella ocellata*, *P. tripartita*, *P. comensis*, *Discostella lacuskarluki, D. pseudostelligera*, and *Pantocsekiella michiganiana*; Benthic diatoms comprised taxa from a variety of genera including small *Fragilaria* (*s*.*l*.) taxa, *Cocconeis*, *Amphora*, *Tabellaria*, *Achnanthes* (*s*.*l*.), *Navicula* (*s*.*l*.), *Diploneis*, *Nitzschia*, *Cymbella* (*s*.*l*.), and *Gomphonema*. GSL: *Cyclotella* (*s*.*l*.) taxa (in order of greatest relative abundance) include *Discostella pseudostelligera*, *Pantocsekiella comensis*, *P. ocellata*, and *P. michiganiana*. *Aulacoseira* taxa (in order of greatest relative abundance) include *Aulacoseira islandica* and *A. subarctica*. Lake Hazen: *Cyclotella* (s.*l*.) taxa (in order of greatest relative abundance) include *Discostella stelligera*, *Pantocsekiella comensis*, and *P. rossii*. Benthic diatoms were almost exclusively small *Fragilaria* (*s*.*l*.) taxa (*Staurosirella*, *Staurosira*, *Pseudostaurosira*).

**Fig. 2.**
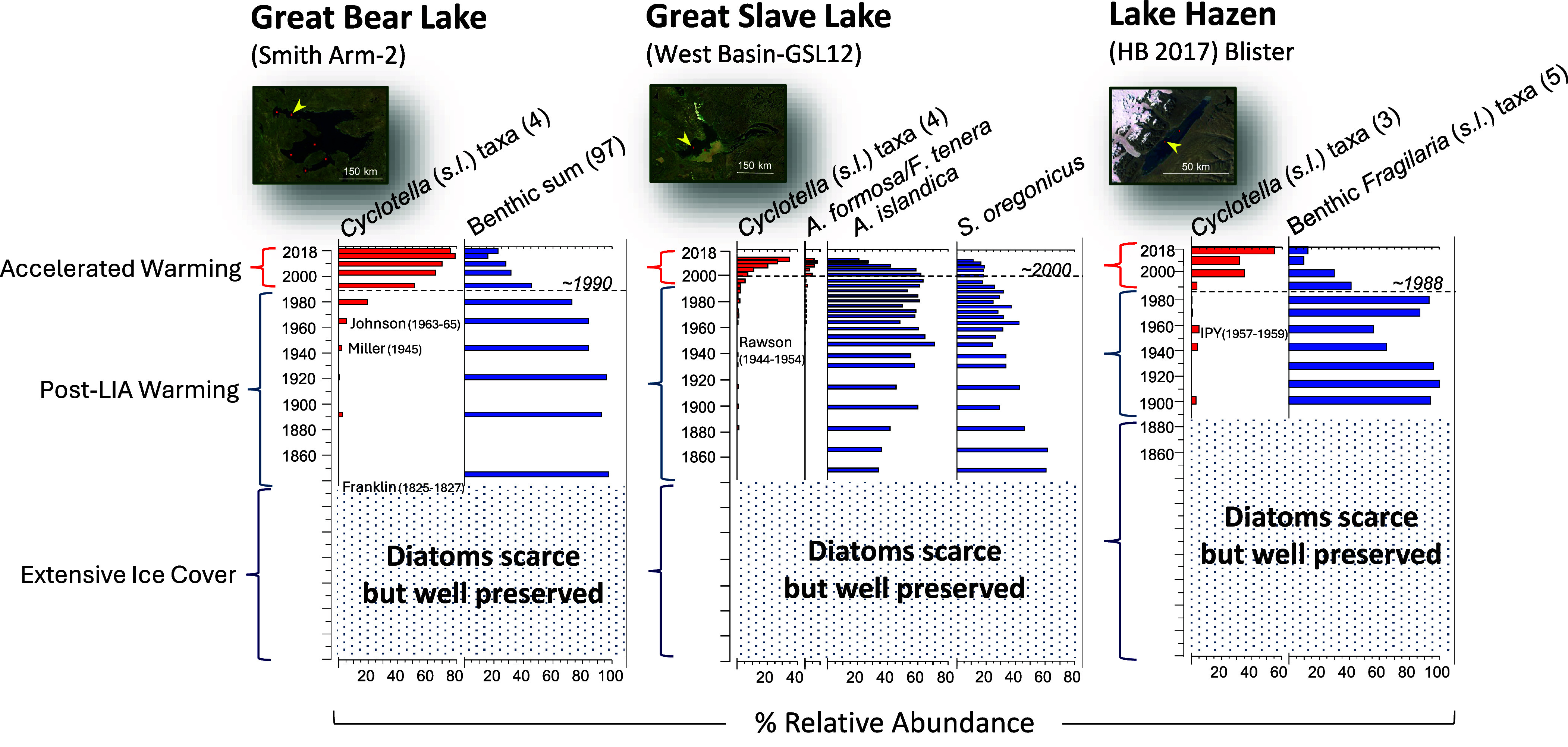
Diatom shifts in sediment profiles representing each of the three “Northern Great Lakes.” Yellow arrow heads in the satellite images point to the coring location on GBL (Smith Arm, site #2), GSL (West Basin, site #12), and Lake Hazen (Blister 2017 site) for the diatom records shown in figure. Dashed horizontal lines represent the greatest diatom compositional shift based on constrained incremental sum of squares (CONISS). Numbers in brackets following taxon names indicate the number of taxa in that grouping. Also included are historical surveys referred to in text. For a more detailed list of diatom taxa in each lake profile, see Fig. 1 caption.

The GBL algal records were remarkably similar in many respects to trends observed in the GSL (23) and Lake Hazen (22) records, including the near absence of diatoms in the oldest sediment intervals, followed by the onset of diatom accumulation post-1850s and a pronounced near-synchronous compositional shift in diatom life-strategy with a rapid rise in euplanktonic diatoms around the turn of the 21st century (Figs. 1 and 2). Below, we discuss similarities and differences in the wholesale ecosystem transformations that these three large northern lakes have experienced during the past ca. 200 y.

### Establishment of Pioneering Diatom Communities.

In the earlier sediment intervals of all lake records, diatoms were too scarce to reliably establish assemblage composition. Notably, there was no evidence of dissolution or other preservation issues, and the rare diatom valves that were encountered were in excellent condition (*SI Appendix*, *Supplementary Text 2*). The paucity of diatoms in preindustrial sediment intervals is commonly reported in large, deep northern lakes that maintain extensive ice cover (9, 22, 23, 63) and corresponds to the Little Ice Age (LIA) ca. 1300 to ca. 1850 CE (64). During this relatively cold period in the Northern Hemisphere, deep and large northern lakes would have experienced even shorter growing periods, low seasonality, and high thermal inertia that would have severely limited open-water diatom growth (3, 7, 10, 23). The establishment of pioneering diatom assemblages in the sediment records of all three lakes signaled the arrival of warmer conditions (post-LIA warming) leading to longer ice-free periods, improved growing conditions, and greater availability of light and nutrient resources (9, 22, 23). Given GBL’s vast size and morphological complexity, variation in the onset of diatom accumulation across lake records was not unexpected, with a delayed establishment at the two deepest sites (Keith-2, McVicar-2) that are closer to the central basin, where ice cover tends to last longer (*SI Appendix*, Fig. S1 and *Supplementary Text 2*). In GSL, the onset of diatom accumulation likewise differed across regions of the lake but, in this case, later onset was mainly associated with the proximity of the sediment record to the Slave River and its massive suspended sediment discharge into the West Basin (23) (*SI Appendix*, *Supplementary Text 2*). In the Lake Hazen records, diatom establishment at the Blister and Main coring locations occurred at approximately the same time (about the turn of the 20th century) (22).

Early diatom assemblage composition was remarkably similar among records within lakes (*SI Appendix*, Fig. S2) but was starkly different across the three lakes (Figs. 1 and 2). Undoubtedly, differences in lake setting, geological history, CA, morphology, the degree and areal extent of ice cover, hydrological inputs (e.g., glacier meltwater, river sediment discharge), water clarity, water residence times, and other site-specific characteristics (*SI Appendix*, Table S1) are important factors affecting algal productivity and composition in the three lakes. In GBL, the early diatom assemblages were characterized by a wide variety (33 to 97 taxa) of benthic diatoms (Fig. 1). Large-celled planktonic *L. bodanica* and *L. affinis* were also important components, whereas small *Pantocsekiella* taxa (*P. ocellata*, *P. tripartita, P. comensis*) were of note, but occurred in distinctly lower relative abundances than in modern intervals (*SI Appendix*, Fig. S2). In Lake Hazen, benthic taxa dominated the early assemblages, but were almost exclusively populated by small, opportunistic benthic fragilarioid taxa (*Staurosirella*, *Staurosira*, *Pseudostaurosira*), a clear departure from the strikingly rich benthic assemblage that characterized the GBL profiles (Fig. 2). The prevalence of benthic taxa observed in the two more northern lakes, GBL and Lake Hazen, is a common characteristic observed in the earlier sediment intervals of numerous Arctic diatom records from deep lakes, where extensive ice cover persists in the pelagic regions, largely limiting diatom production to the near shore benthic environments (3, 10, 22). In contrast, the early diatom assemblages of GSL were strongly dominated by large filamentous *A. islandica* and large centric *Stephanodiscus oregonicus*, while benthic diatoms and cyclotelloid taxa were extremely rare (Figs. 1 and 2). The dearth of benthic diatoms in the GSL records would be expected given the large fetch, strong wave action and sediment resuspension, and the highly turbid, light-limited conditions of the West Basin where our cores were collected (23).

Although growing conditions improved with 19th century climate amelioration, it undoubtedly remained a challenging environment for diatoms, particularly in the more northerly GBL and Lake Hazen, as can be attested by historical limnological reports of extremely low productivity. For example, in 1945, almost a century after the end of the LIA, Miller described net plankton collected from GBL as being exceedingly meager (42), whereas in Lake Hazen, McLaren remarked on the virtual absence of phytoplankton in the net hauls taken in the summer of 1958 (35). In GSL, Rawson observed that the plankton crop (dominated by diatoms) that he calculated for the West Basin during the 1940s (25.1 kg/ha) was over 50% higher than what he calculated for GBL (12 kg/ha) from three vertical net hauls collected by Miller in 1945 (24). Although details of these early GBL samples are sparsely reported (24, 56), Rawson highlighted similarities to extremely deep, ultraoligotrophic Christie Bay in the East Arm of GSL including low mineral content, low plankton crop (14.3 kg/ha), high water clarity, as well as comparable dominant plankton forms (diatoms: *Asterionella formosa*, *Tabellaria fenestrata*; rotifers: *Keratella*, *Kellicottia*; copepods: *Diaptomus*, *Cyclops*, *Limnoclanus*) (24). Much like Rawson’s evaluation of the East Arm of GSL, these findings supported Miller’s assessment that GBL could not sustain a large-scale commercial fishery: later limnological surveys on GBL by Johnson (44) reached the same conclusion.

While both GBL and Lake Hazen are deep and large and had low productivity during the early diatom period (24, 35, 42), they differ substantially in several key limnological features (*SI Appendix*, Table S1) that could account for differences in the composition of pioneering diatom assemblages. For example, observations during the 1957 to 1962 surveys of Lake Hazen found that brief ice-free periods were typically confined to a shallow, nearshore moat (21, 50, 51), and muddy glacier-fed riverine inputs led to turbid underflows (34, 35). Despite Lake Hazen’s great depth, the brief and highly turbid ice-free periods would have prohibited the establishment of open-water (planktonic) diatoms and restricted benthic taxa to species that can tolerate these severely limited light and nutrient resources (22). Opportunistic benthic *Fragilaria s.l*. species that dominated the early assemblages of the Lake Hazen records meet these requirements as they tend to compete well in the challenging conditions associated with extensive ice cover (65) and minerogenic turbidity (66). By comparison, the more protected arms of GBL, where early surveys reported waters to have been warmer with local areas of comparatively higher productivity than in the main body of the lake (42, 44), would have experienced less severe conditions for diatom growth than High Arctic Lake Hazen. Moreover, GBL is not glacier-fed and with only a few major tributaries, lends to its exceptionally high water clarity that has been emphasized in historical reports for at least the past 200 y. For example, Sir John Richardson (Franklin expedition of 1825–1827) observed that a white rag lowered into the water did not disappear until a depth of 15 fathoms (27 m) (41). This rudimentary (but ingenious) transparency determination taken 40 y before the Secchi disk was invented (1865) was remarkably similar to measurements taken over a century later by Miller in 1945 (29 m) (42) and by Johnson in 1963 (30 m) (44, 45). Such high water clarity, together with a relatively greater ice-free extent at the height of the summer, would have permitted the establishment of a diversity of nearshore habitats and hence a much richer variety of benthic diatoms (including epiphytic taxa) compared to Lake Hazen’s depauperate pioneering assemblage (Figs. 1 and 2 and *SI Appendix*, Fig. S2). Additionally, the high transparency and brief ice-free periods in the pelagic regions of GBL’s southern arms may have provided adequate growing conditions for open-water diatoms. This may explain why GBL was the only lake in our study where planktonic cyclotelloid taxa occurred in the earlier sedimentary assemblages (Figs. 1 and 2), particularly larger-celled *Lindavia bodanica* and *L. intermedia* (*SI Appendix*, Fig. S2). Similarly, in an early paleolimnological study of the very deep and clear McLeod Bay in the East Arm of GSL, Stoermer et al. (63) noted that planktonic diatoms contributed substantially to the assemblage during the preindustrial era, especially large *Lindavia* taxa.

Interestingly, filamentous *Aulacoseira* taxa, which were so prominent in our GSL cores, were conspicuously scarce in the GBL and Lake Hazen sediment records (Figs. 1 and 2 and *SI Appendix*, Fig. S2). In the West Basin of GSL, *A. islandica* was by far the dominant phytoplankter of all the algal species reported in historical limnological surveys undertaken between the 1940s and 1990s (24, 48, 49). This heavily silicified diatom requires turbulent mixing, high silica and mineral content, can tolerate low light levels, and may even bloom under ice during winter circulation in large, cold lakes (24, 67). These conditions characterize the West Basin where the strong influence of the Slave River brings in relatively warm, turbid, productive waters with a massive delivery of nutrient and mineral-rich suspended sediments (18) that collectively attribute to the success of this taxon (23). In stark contrast*, Aulacoseira* establishment in GBL and Lake Hazen would have been impeded by extremely clear, mineral-poor, ultraoligotrophic conditions as well as by the perennially frozen offshore regions in Lake Hazen. These same challenging growing conditions characterize the very deep East Arm of GSL, where *Aulacoseira* taxa were rarely observed in historical (24) and paleolimnological (63) records and assemblages were comprised of a variety of benthic taxa and large-celled *L. bodanica*, comparable to GBL. Indeed, Rawson examined three 1945 vertical plankton hauls from GBL sent to him by Miller and concluded that the amount and composition of plankton (including dominant diatoms) were similar to those he observed in Christie Bay of GSL, particularly noting the scarcity of *Aulacoseira* species (24). Lund (47) later examined the periodicity of *A. islandica* using Rawson’s 1940s net samples from the West Basin where he remarked on the potential importance of this large-celled diatom to the rest of the GSL food web and, ultimately, for sustainable fisheries. The near absence of *Aulacoseira* taxa in our GBL and Lake Hazen sediment records is undoubtedly a testament to a combination of short, cold growing seasons, extreme oligotrophy, low mineral content, and low productivity (particularly in the open-water regions) of these systems. These same extreme conditions are what led Miller and Rawson to conclude that a commercial fishery was not viable in GBL (42) nor in the East Arm of GSL (24).

### Pronounced Reorganization of Algal Assemblages at the Turn of the 21st Century.

Despite considerable differences in latitude, limnological features, and in diatom founding populations, the three lakes registered strikingly similar and largely synchronous compositional shifts near the turn of the 21st century (Figs. 1 and 2). In all sediment records, this assemblage reorganization was marked by higher relative abundances of small-celled (often <3.0 µm), euplanktonic cyclotelloid taxa (*Pantocsekiella* *ocellata*, *P. tripartita, P. comensis*, *P. gordonensis*, *P. michiganiana*, *P. rossii*, *Discostella lacuskarluki, D. stelligera*, *D. pseudostelligera*) (Figs. 1 and 2 and *SI Appendix*, Fig. S2). In GSL (23) and, in the majority of the GBL cores, the increase in cyclotelloid taxa was accompanied by increases in elongate planktonic taxa (*Asterionella formosa*, **Fragilaria* nanana*, **F*. tenera*, *Urosolenia*, *Tabellaria flocculosa* str. III, *Ulnaria acus*) (Fig. 2 and *SI Appendix*, Fig. S2).

**Fig. 3.**
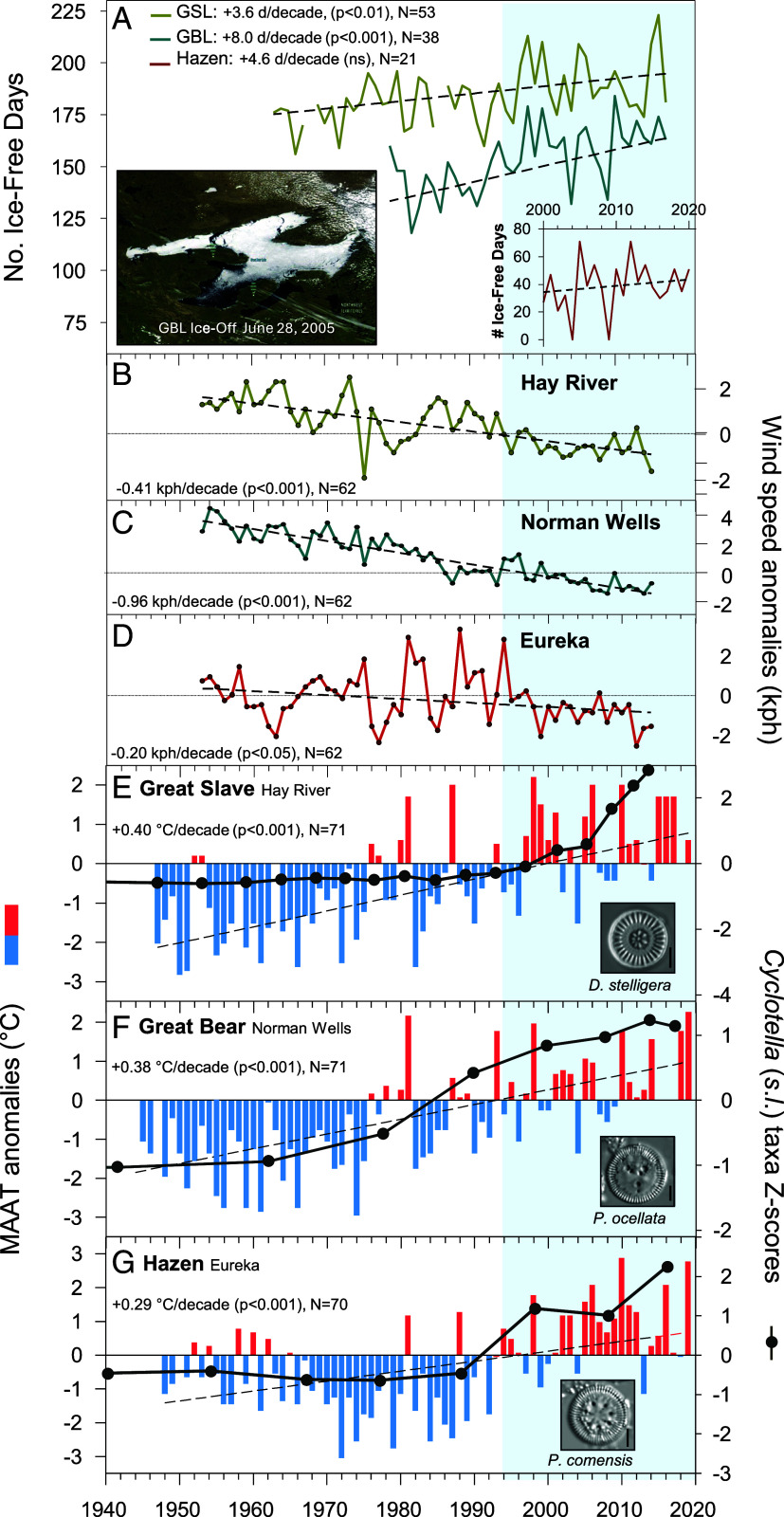
Synthesis of climate and paleoproxy trends from GBL, GSL, and Lake Hazen over the period of the instrumental records (1940–2020). (*A*) The number of ice-free days: GSL based on a 54-y composite record combining Hay River ground observations (1963–1991) with remote sensing data (1992–2017) from Su et al. (52). GBL, near Keith Arm based on remote sensing (1980–2017) data from Su et al. (52). Lake Hazen (*Inset*) data derived from NASA daily satellite images available since 2000 (provided by D. Muir). Inset: satellite image (NASA) of typical ice-off progression on GBL highlighting differences in timing across arms. (*B***–***D*) Mean annual wind speed anomalies from climate stations near GSL (Hay River) (*B*), GBL (Norman Wells) (*C*), and Lake Hazen (Eureka) (*D*). (*E* and *F*) Mean annual air temperature (MAAT) anomalies (red and blue bars) and changes in the percent relative abundance (plotted as z-scores) of *Cyclotella sensu lato* (*s*.*l*.) taxa (line with black circles) for GSL-12 (*E*), GBL, Smith-2 (*F*), and Lake Hazen, Blister (*G*). Wind speed and MAAT anomalies data were based on a 1981–2010 baseline. Panels *A*–*G* include Mann–Kendall test and Sen’s slope estimator (dashed lines) results that were used to calculate the significance of monotonic trends and magnitude of changes in the climate data. Light micrographs in *E*–*G* represent the most common cyclotelloid taxa in the lake records. (Scale bar, 3 µm.) Shading represents period of accelerated climate change (mean breakpoint for MAAT and wind speed across stations = 1994) when cyclotelloid taxa proliferated. ns = not significant.

**Fig. 4.**
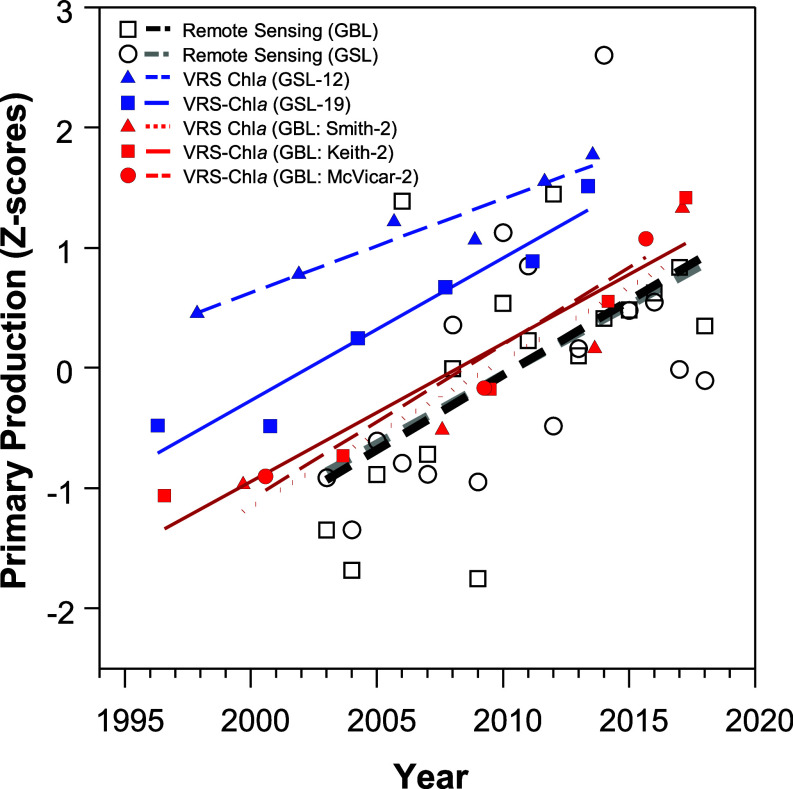
Recent increasing trend in primary production in GBL and GSL. Comparison of changes in primary production measured by visible range spectroscopy-inferred chlorophyll-*a* (VRS-Chl*a,* this study) and by remote sensing (Sayers et al.; ref. 14) for GSL and GBL. Linear trendlines are shown as dashed and solid red (GBL) and blue (GSL) lines for this study (*P* < 0.0001) and as dashed black (GBL) and gray (GSL) lines for the remote sensing (14) (*P* < 0.05). All data were converted to *z*-scores to facilitate comparisons.

This diatom compositional shift occurred during the period with the lowest wind speeds and highest air temperatures recorded at the Norman Wells (GBL), Hay River (GSL), and Eureka (Lake Hazen) weather stations, as well as during the longest ice-free periods registered at each lake (Fig. 3). Significant monotonic trends (Mann–Kendall test) were evident for all climate data (except for the relatively short Lake Hazen ice record), although the magnitude of change (Sen’s regression) varied among lakes (Fig. 3 *A*–*G*). The increase in the number of ice-free days was greatest in GBL (8.0 d/decade near Keith Arm since 1980, *P* < 0.001), followed by Lake Hazen (by 4.6 d/decade since 2000, not statistically significant), and lower in the West Basin of GSL (3.6 d/decade since 1963, *P* < 0.01) (Fig. 3*A*). Mean annual wind speed anomalies showed significant declines at all weather stations, particularly since the 1990s, with the greatest decline recorded at Norman Wells (0.96 kph/decade, *P* < 0.001), followed by Hay River (0.41 kph/decade, *P* < 0.001) and the lowest at Eureka (0.20 kph/decade, *P* < 0.05) (Fig. 3 *B*–*D*). Initially climate data from Alert were considered for comparisons, but both air temperature and wind speed trends at Eureka best matched available shorter-term instrumental records from Lake Hazen and Tanquary Fiord airport (*SI Appendix*, *Supplementary Methods* and Table S3) as the Hazen basin is known for its unusual wind regime (68). Mean annual air temperature (MAAT) anomalies significantly increased (*P* < 0.001) at all weather stations (Fig. 3 *E*–*G*), with similarly high rates of change recorded at Hay River (0.40 °C/decade) and Norman Wells (0.38 °C/decade) and relatively lower changes at Eureka (0.29 °C/decade); however, all three records were well above the global average MAAT anomalies (0.08 °C/decade). MAAT was distinctly cooler than average in the earlier parts of all records (negative bars) whereas following the 1990s, most years were warmer than average (positive bars) at all weather stations (Fig. 3 *E*–*G*). It was during this warmer period that cyclotelloid taxa started to increase in all lake records, becoming more pronounced after ca. 2000 (Figs. 2 and 3 *E*–*G*), accompanied by relative declines in once dominant taxa, including benthic diatoms in GBL and Lake Hazen and large filamentous *A. islandica* in GSL (Fig. 2 *A*–*C*). Accompanying the shift in diatom community composition, our paleolimnological records also registered an increasing trend in lake-wide algal production in GBL and GSL (VRS-Chla; *P* = 0.05 to 0.0003) that was an excellent match with increases in primary production in these two lakes measured by remote sensing (GSL *P* = 0.028, GBL *P* = 0.016) (14) (Fig. 4).

Declining trends in lake ice cover in GBL, GSL, and Lake Hazen (Fig. 3*A*) align with ongoing survey, observational, and lake monitoring data that have reported recent reductions in ice thickness (69, 70) and shorter ice-cover periods (21, 39, 52, 71). On GBL, local observations indicate that the timing of ice-off has shifted a remarkable 3 wk earlier over the past 30 y (11). Only ~50 y ago, High Arctic Lake Hazen only occasionally experienced ice-free conditions, but now perennial ice cover is rare, and open water is common between late July and early August (21), when there is 24-h daylight. In GSL, the ice-free period in the West Basin has lengthened by almost 3 wk in ~50 y (1963–2017), with ice-off occurring earliest at locations nearest the Slave River (23, 52). The degree and speed of lake ice phenology changes observed in the study lakes align with changes in water column properties including recent surface temperatures (33, 72) that are warmer than historical readings (44), and weaker vertical mixing, increased thermal stability and/or longer and stronger periods of summer thermal stratification (11, 27), all of which affect algal resources including light and nutrients (2, 5).

The recent climate-mediated changes in fundamental limnological properties and aquatic ecosystem processes that the three lakes have experienced (Fig. 3 and *SI Appendix*, *Supplementary Text 3* and *Supplementary Methods*) provide ideal conditions for the proliferation of smaller-celled *Cyclotella s*.*l*. taxa and elongate planktonic taxa (5) observed in our 21st century sediment records. These euplankton have high surface area to volume ratios that would reduce sinking velocity (73) and may facilitate efficient nutrient uptake and light harvesting (74). A warmer and more thermally stable water column would favor these ecophysiological traits that characterize the modern diatom assemblages over the previously dominant benthic diatoms (65) and the heavier, fast sinking *Aulacoseira* taxa (75). The displacement of long-established diatom assemblages by fast-growing, small-celled cyclotelloid taxa is a well-documented response to warming that has been reported in hundreds of small to mid-sized lakes worldwide (5, 6, 76). In deep, clear McLeod Bay on the East Arm of GSL, an increase in small cyclotelloid taxa in the top few centimeters of a sediment core collected in the 1980s by Stoermer et al. (63) likely represents the initial stage of this shift for the rest of the lake. In the Laurentian Great Lakes (LGL), sediment records registered similar diatom compositional shifts (starting around the early 1970s) but, unlike the comparatively unpolluted large lakes in the remote Arctic, these more southern lakes have been subjected to a long history of multiple environmental stressors, confounding the simultaneous effects of recent warming (77). Since the turn of the 21st century, these same diatom compositional shifts are now clearly expressed in three of Canada’s largest and deepest pristine northern lakes, signaling a critical reorganization in algal community structure.

### Is There Evidence for a Lake-Wide Shift in GBL’s Mictic State?

GBL has long been identified as a freely circulating, cold monomictic lake (62) based largely on key limnological surveys by Johnson during the 1960s (43, 44, 78). For example, historical water column temperature data collected annually in July and August from 1963–1966 in the colder and deeper regions of the main basin (six miles west of Port Radium in McTavish Arm) documented essentially isothermal (~4 °C) conditions (43, 44, 78). However, this classification may no longer apply for the entire lake (33). Water column temperature profiles, collected since 2014 at approximately the same time of year and location on McTavish Arm that Johnson sampled 50 y earlier, show a departure from its long-reported isothermal and monomictic character, with some years now exhibiting a well-developed summer thermocline and surface temperatures exceeding 10 °C. In the more protected McVicar, Keith, and Smith arms, monitoring data collected since 2012 (11, 27) and in summer 2008 on Keith Arm near Délınę (33) likewise showed persistent summer thermal stratification with well-developed thermoclines. Extending the limnological records back further in time, our diatom profiles suggest that, since around the turn of the 21st century, summer thermal stratification in these arms is a recent development (or at some sites, is now more strongly developed). However, “ground-truthing” our paleolimnological interpretations in the more protected and warmer arms of GBL provided a challenge, as historical temperature records in these regions of the lake are exceptionally scarce. Nevertheless, information gleaned from rare (often incidentally reported) historical temperature measurements corroborated our paleoecological data (*SI Appendix*, *Supplementary Text 3*). For example, Kennedy (79) and Miller and Kennedy (56) provided evidence that, during their 1945 survey, most parts of GBL were oligotrophic and likely did not thermally stratify during the summer (or at least not strongly), with exceptions being the most isolated and shallower bays such as the southern-most tip of McVicar Arm. In his report on Coregonine fish, Kennedy (79) stated that the average 1945 summer surface water temperature in Keith Arm (near Délınę where Miller and Kennedy set up camp) was only slightly higher than temperatures recorded at the end of July of that year near Port Radium in McTavish Arm (~6 °C; ref. 42). This is consistent with the August 1945 mean water temperatures across GBL reported by Larkin (62) of ~5 °C with occasional recordings of temperatures above 7 °C in late August at locations deeper than 30 m: no summer thermocline was observed, and thermal stratification was rare or completely absent. A few decades later, Johnson mapped the distribution of surface water temperatures across GBL in August 1963 and 1964 (44) and found they were consistently below 10 °C (except for the southernmost tip of McVicar Arm) at locations proximal to where our six sediment cores were collected (*SI Appendix*, *Supplementary Text 3*). More recently, August measurements taken in 2008 (33) and since 2012 (11, 27, 72) at approximately the same locations on Smith, Keith, and McVicar arms indicate that surface water temperatures now typically exceed 10 °C. The relatively cool surface water temperature readings from the 1940s (56, 62, 79) and the 1960s (44) suggest it was unlikely that summer thermal stratification developed in the southern arms of GBL in the past, and, if it did, it would have been weak and likely not persistent.

### Whole Lake Implications and Conclusions.

The near-synchronous shift in diatom composition across these three “Northern Great Lakes” is a hallmark threshold-like response to recent accelerated warming and associated water column changes (5), especially in relatively pristine lakes such as these, with no history of nonindigenous species introductions (19, 26). In GBL, early limnological accounts substantiate our finding that the transition from a previously cold monomictic state to a new thermal regime is a very recent and lake-wide phenomenon. The recent development of a deep chlorophyll maxima in GBL (11) is further evidence of a change in lake mictic state, with recent increases in nutrient accumulation and phytoplankton production that are predicted to have consequences on food distribution and prey availability for the lake’s cold-adapted fish species [(11, 55), *SI Appendix*, *Supplementary Text 4*]. In Lake Hazen, historical surveys from the 1950s–1960s (34–36) designated the lake as an amictic to cold monomictic system that rarely experienced completely ice-free conditions (21), whereas surveys of GSL’s West Basin during the 1940s reported sporadic, short-lived, but well-developed summer thermal stratification in some years (24, 62). However, with accelerated warming and declines in wind speed in recent decades, Lake Hazen’s amictic character is eroding as ice-free summers are now becoming the norm (21), while the dimictic structure of the West Basin of GSL has strengthened (23). In addition, over the past ~15 y, there has been an unprecedented rapid increase in the frequency and spread of cyanobacteria blooms in GSL, likely promoted by warmer waters and calmer winds (25). These rapid transformations in the physical and thermal regimes of these large, relatively pristine lakes indicate that lake warming is a dominant stressor (11) and is likely the main driver of the shifts in primary producers observed in our sediment records.

From an aquatic food web perspective, large-scale changes in algal composition and aquatic primary production, in response to fundamental alterations in lake water properties, are forerunners of an entire lake ecosystem transformation (7, 80). It is important to obtain a better understanding of how recent climate-related transformations in lake properties and changes at the base of the food webs of these large, remote northern lakes will affect the growth and health of zooplankton and smaller plankton-grazing forage fish, as this will ultimately have repercussions on top predators and key harvested fish species (*SI Appendix*, *Supplementary Text 4*). This will require continued and intensive monitoring and research which should include examining the transfer of food and energy between different organisms up the food web. One might reason that an overall increase in lake-wide primary production would result in a greater quantity of food available to higher trophic levels. However, the compositional shift to small, fast-growing, cyclotelloid diatoms, that are often negatively selected by zooplankton predators (81), has likely reduced the quality of essential dietary micronutrients that are synthesized by primary producers and then transferred to consumers at higher food web levels (81–85). For example, in marine systems, climate-associated shifts to smaller-sized algae (86) were found to affect diatom production of omega-3 long-chain polyunsaturated fatty acids, an essential nutrient for maintaining a healthy energy flow throughout the entire food web (83, 84).This may be of particular importance in large, deep northern lakes, where primary production is generally low and phytoplankton play a critical role in ecosystem functioning and food web dynamics (11, 24, 47, 55). Indeed, in GBL, lake trophic network analysis using DFO, SRRB, and DRRC lake survey data identified phytoplankton as one of the key drivers of the lake’s relatively simple food web, providing an estimated 55% of total energy flow to upper trophic levels including zooplankton, Cisco and Lake Trout (11). These findings are supported by combined stomach content and fatty acid analyses of GBL Lake Trout tissue, which indicate biomarker signatures characteristic of diatoms (including pennate taxa) and calanoid copepods (87). This reflects trophic transfer through the food web via zooplanktivorous Cisco and *Mysis sp*., both of which are key prey resources for Lake Trout (87). Therefore, a shift to smaller-sized cyclotelloid diatoms will likely impact the diets of plankton feeders such as large crustacean zooplankton (mysids and amphipods) that, together with calanoid copepods, have long been important macroinvertebrates in GBL and GSL (62), dominating the trophic flow at intermediate food web levels (11, 55). Meanwhile, in Lake Hazen, estimating the impact that a shift to small-celled planktonic diatoms will have on the lake’s only fish species, Arctic Char (*Salvelinus alpinus*), is further complicated by the recent rise in glacial runoff and minerogenic turbidity that has already compromised this visual predator’s ability to find its prey (21). However, as the ice-free area and diatom production expands beyond near-shore moat environments (3, 7, 10, 22), the increase in planktonic diatoms in our sediment records tentatively complements the evidence for a recent shift toward more pelagic feeding habits of Lake Hazen Arctic Char (88), (*SI Appendix*, *Supplementary Text 4*).

In the West Basin of GSL, small, euplanktonic diatoms are quickly replacing long-established, large-celled *Aulacoseira* species (23), that Lund (47) suggested were an important food source for upper trophic levels in GSL and other northern Canadian lakes. Indeed, similar diatom compositional shifts reported in the more southern LGLs (77) led to a shift in diet of a key benthic crustacean in southern Lake Michigan (81). Here, a pronounced decline in calorie-rich *Aulacoseira*, concurrent with an increase in nutrient-poor, small cyclotelloid taxa, played a major role in the functional extinction of the amphipod, *Diporeia*, with well-documented consequences for the entire food web (ref. 81, *SI Appendix*, *Supplementary Text 4*). By this reasoning, the recent diatom compositional shifts observed in our paleolimnological records may have a substantial impact on food transfer in GSL, where large, lipid-rich *Aulacoseira* taxa have undergone rapid declines in recent decades. Nevertheless, the nature of the fundamental changes we report are trending toward a degradation in the pathways of energy flow through these large northern systems. Paleolimnological data such as these provide important context for these rapidly changing aquatic ecosystems as well as supply valuable benchmarks for the continued stewardship of three of the world’s largest pristine lakes.

In summary, the marked restructuring of algal communities in three of the world’s largest and deepest Arctic lakes is a clear sign that even some of the most climate-resistant freshwater bodies have now succumbed to climate warming and have diverged from their 20th century conditions. Under the new climate regime, the continued collection and analysis of detailed neolimnological data will be required to better understand trophic interactions. The full effects of these key compositional changes in the lakes’ primary producers and consumers are currently unknown but will almost certainly affect overall energy flow and cascade throughout the food web.

## Methods

### Sediment Coring and Water Sampling.

Six GBL sediment cores were retrieved in 2018 by Environment Canada and Climate Change (ECCC) from three arms (Smith, Keith, McVicar) of the lake providing a spatially extensive representation (65.1°N to 68.8°N, *SI Appendix*, Fig. S5) of the peripheral regions of GBL (*SI Appendix*, *Supplementary Methods*). GSL sediment cores were collected in 2014 by ECCC from two offshore locations in the West Basin (GSL12 and GSL19; ref. 23). All sediment cores for GSL and GBL were subsampled into 0.5 cm contiguous intervals for the upper 20 cm, and every interval containing sufficient valves was enumerated. Lake Hazen sediment cores were collected in 2017 from the Blister site and in 2013 from the main basin site and subsampled into 0.5 cm contiguous intervals throughout the cores (22). Comparisons of key limnological characteristics among the lakes (*SI Appendix*, Table S1) were based on data collected from various sources including peer reviewed publications, government reports, and field notes (GBL). For GBL, water column measurements and lake water samples were collected on the same day and at the same location as the sediment core, the latter were submitted to the National Laboratory for Environmental Testing (NLET) in Burlington, Ontario for analyses (*SI Appendix*, Table S2).

### ^210^Pb Dating.

Chronologies for GBL, GSL, and Lake Hazen sediment cores were established using gamma spectrometry via high-purity germanium, well-type detectors (DSPEC, Ortec®) at PEARL, Queen’s University that determined radioisotope activities for ^210^Pb, ^214^Pb (or ^214^Bi), and ^137^Cs (GBL results can be found in *SI Appendix*, *Supplementary Methods* and Figs. S5 and S6; for GSL dating results see ref. 23; for Lake Hazen, see ref. 22).

### Diatom Analysis.

Sediment sample processing and diatom taxonomy and enumeration followed standard procedures and protocols used at PEARL (*SI Appendix*, *Supplementary Methods*).

### Spectral Analyses.

Visible range spectroscopy-inferred chlorophyll-*a* (VRS-Chla) measurements were used to examine temporal trends in lake primary production following a log-transformed algorithm of standardized methods (89) that capture the summed concentration of all compounds associated with primary Chl-*a* isomers, as well as the main degradation products, pheophytin *a* and pheophorbide *a*. Comparisons were made to the Sayers et al. (14) primary productivity data for GBL and GSL derived from remote sensing and representing the photic zone averages for the ice free period of each year (*SI Appendix*, *Supplementary Methods*). VRS-Chla concentrations for Lake Hazen sedimentary records were too low to register trends.

### Weather Station Records.

Historical air temperature and wind speed data obtained from Environment Canada [adjusted and homogenized climate data (ahccd)] and from Parks Canada (for Lake Hazen) weather stations were critically assessed (*SI Appendix*, *Supplementary Methods*, Table S3, and Figs. S7–S9) prior to selecting the Norman Wells (GBL), Hay River (GSL), and Eureka (Lake Hazen) weather stations to provide the most representative and continuous data for each lake location. Piecewise linear regressions were used to identify significant points of change (breakpoints) in both MAAT and wind speed data from each climate station (*SI Appendix*, *Supplementary Methods*). The mean breakpoint for wind speed and MAAT across the stations was used to represent the period of accelerated change in the instrumental records. To facilitate comparisons between instrumental records and paleolimnological data, all datasets covered similar time periods between 1948 and 2019. For all stations, MAAT and mean annual wind speed data were presented as anomalies data calculated from the same 30-y period (1981–2010 mean). Global MAAT anomalies also covered similar time periods and were calculated from the 1981–2010 mean.

### Ice Phenology Records.

Trends in the number of ice-free days for the three lake regions were based on ground observations [GSL, the National Snow and Ice Data Center (NSIDC) (ref. 90; https://doi.org/10.7265/N5W66HP8)], passive microwave remote sensing data (GBL and GSL; ref. 52), and from examining and comparing NASA MODIS daily satellite images (GBL, Lake Hazen) (*SI Appendix*, *Supplementary Methods*, Tables S3 and S4, and Figs. S10–S12).

For historical time series data (air temperature, wind speed, number of ice-free days) a Mann–Kendall test was applied to determine statistically significant monotonic trends, whereas Sen’s slope estimator was used to calculate the direction and magnitude of change using MAKESENS 1.0 software (https://www.researchgate.net/publication/259580998) (91).

## Supplementary Material

Appendix 01 (PDF)

## Data Availability

Diatom and radioisotopic data for the 10 sediment records (GBL, GSL, Lake Hazen) are available on the Neotoma Paleoecology Database: Great Bear Lake (Smith-1): https://doi.org/10.21233/ng92-k602 (92). Great Bear Lake (Smith-2): https://doi.org/10.21233/ntfs-k706 (93). Great Bear Lake (Keith-1): https://doi.org/10.21233/js3h-5176 (94). Great Bear Lake (Keith-2): https://data.neotomadb.org/74136 (95). Great Bear Lake (McVicar-1): https://doi.org/10.21233/hpe7-gy31 (96). Great Bear Lake (McVicar-2): https://doi.org/10.21233/e9ey-vc78 (97). Lake Hazen (Blister): https://doi.org/10.21233/jccz-sk46 (98). Lake Hazen (Main): https://doi.org/10.21233/7mzd-s343 (99). Great Slave Lake (GSL12): https://doi.org/10.21233/z37z-wd37 (100). Great Slave Lake (GSL19): https://doi.org/10.21233/3mx8-vs75 (101). All other data are included in the manuscript and/or *SI Appendix*.
