# Supplementary material for: New climate regime restructures the ecology of Canada’s Northern Great Lakes
Source: Proc Natl Acad Sci U S A. 2026 Jun 1;123(24):e2603271123. doi: 10.1073/pnas.2603271123 (PMC13273309; doi:10.1073/pnas.2603271123)
Supplement: Supplementary file 1 — Appendix 01 (PDF) [file pnas.2603271123.sapp.pdf]

PNAS

Supporting Information for

New climate regime restructures the ecology of Canada's 'Northern Great Lakes'

Kathleen M. Rühland[1]*, Neal Michelutti[1], Marlene S. Evans[2], Kimberly L. Howland[3], and John P. Smol[1]*

[1]Department of Biology, Paleoecological Environmental Assessment and Research Lab (PEARL), Queen's University, Kingston, ON, K7L 3N6, Canada

[2]Environment and Climate Change Canada, Saskatoon, SK, S7N 3H5, Canada

[3]Arctic Fisheries and Marine Mammal Science Division, Freshwater Institute, Fisheries and Oceans Canada, Winnipeg, MB, R3T 2N6, Canada

* To whom correspondence may be addressed

Email: smolj@queensu.ca OR ruhlandk@queensu.ca

This PDF file includes:

- Supplementary Text 1 to Supplementary Text 4
- Supplementary Methods
- Figures S1 to S12
- Tables S1 to S3
- SI References

Supplementary Text 1

‘Northern Great Lakes site descriptions, historical information and comparisons

Table S1: Comparison of lake characteristics among the three ‘Northern Great Lakes’

	GREAT BEAR LAKE	GREAT SLAVE LAKE	LAKE HAZEN
Latitude (°N)	65.9	61.5	81.1
Setting	Arctic Circle	Sub-Arctic	High Arctic
Depth Max. (m)	446	614	267
Depth Mean (m)	71	69	85
Lake Surface Area (km^2)	31,326	28,568	540
Catchment Area (km^2)	145,870	971,000	6860
Catchment Area: Lake Area	4.7	33.9	12.7
Lake Volume (km^3)	2236	1580	51.4
Catchment Area:Lake volume (m^2m^3)	65.2	615	95
Mixing Type/Lake Circulation (historical)	Cold monomictic and Isothermal[1]	Dimictic	Amictic to cold monomictic
Open Water (# days)	~153 (variable)	~175 to 184	some years zero
Water Residence Time (yr)	124	7 to 16	25 [2]
Secchi Depth (m)	~30 CLEAR	~2.5[3] TURBID	1.7 to 8.32[4] TURBID
Lake Temp. (°C) surface waters in August	~4	~10	~2
pH	~8.0	~8.0	~7.8- 8.2
Total Phosphorus (µg/L)	1.7 to 4.1 [5]	~6 to 25	<3.0
Primary Production (mg C/m^2/Day)	(99.8[6]) to 183 [7]	286 [7]	too low to measure [8]
Fishery (Commercial)	NO	YES	NO
Fisher (Subsistence, Sport)	YES	YES	Minimal
Population Living Near Shoreline (2021 census)	~800	~27,263	None

1. However, 2012-2023 monitoring data indicate most of GBL now exhibits persistent thermal stratification and development of summer thermocline (1, 2).

2. Historically ~89 years (3) but ~10-fold increase in glacial meltwater since ca. 2007 reduced water residence time to ~25 years (4).

3. High turbidity in West Basin of GSL where Slave River sediment input has strong influence on water clarity.

4. Spring versus summer readings in 1958 (5). Summer has higher turbidty than in spring in Lake Hazen as a result of inflow from melting glaciers.

5. Based on March 2018 measurements from two locations on three arms (Smith, Keith, McVicar) by ECCC.

6 Based on 2012-2018 measurements from ref. 6

7. Estimated from remote sensing data 2003-2018, published in Sayers et al. (7).

8. Based on mean July - August data 1957-1958 and a Secchi depth of 8.3 in clear water(5). Recent data do not exist.

Great Bear Lake (GBL) additional information

Ice off on GBL varies across the lake typically moving from southeast to northwest, first occurring on McVicar Arm, progressing to Keith Arm and then to Smith Arm (see example from 2005 in Figure S1 below). Radium and uranium mines were in operation between 1932 and 1962 (Port Radium on McTavish Arm) and later serviced silver mining activities until closing permanently in 1975 (8). Despite its relatively brief mining history, GBL is considered to be the most pristine example of a truly large, cold, freshwater ecosystem (6, 8, 9). Indeed, during the 1960s, Johnson (10) referred to GBL as being in much the same condition as when John Franklin visited in 1825.

Comparison of key lake characteristics among three lakes

Among freshwater lakes worldwide, GBL, GSL and Lake Hazen are among the 253 lakes that have a surface area exceeding 500 km^2 and among the 75 lakes that have a maximum depth greater than 100 m (11, 12) (Table S1). Key lake characteristics for comparisons among GBL, GSL and Lake Hazen were collected using available data from various sources including peer reviewed publications, government reports and field notes (Table S1).

Historical surveys

Historical reports relevant for comparisons to our paleolimnological study are rare but do exist for primary production (GBL: (13, 14); Lake Hazen (5, 15); GSL (14, 16)) and phytoplankton (numbers and composition) (GBL (14, 17); Lake Hazen (5); GSL (14, 16, 18). Even more rare but of great interest are historical diatom collections from these lakes including for GBL (1976-1978) by Moore (17) and the West Basin of GSL (1944 to 1954) by Rawson (14), open water season of 1986 by Fee et al. (16), and in March 1994 by Evans (18). The earlier silk net tows (~76 µm mesh equivalent) used by Rawson on GSL (14) and Miller on GBL (15) to estimate primary production would not have captured smaller size ranges (picoplankton, nanoplankton) phytoplankton and therefore these estimates of primary production are undoubtedly an underestimate). Indeed, Fee et al. (16) noted that the absence of small phytoflagellates in GSL was unquestionably an artefact of nets used to collect samples and that these flagellates were as abundant then as the were when they undertook sampling in the 1970s. Nevertheless, these earlier estimates are of interest, particularly for comparing values across lakes that used the same methodologies during the 1940s and 1950s. No historical diatom collections were made for Lake Hazen, although primary production and plankton research were undertaken between 1957 and 1958 (with some additional measurements in 1961 to 1963) (5, 15) as part of the International Geophysical Year (IGY). For example, McLaren (5) noted that primary production values in the open water of Lake Hazen on August 9 to 11 of 1958 were too low to be measurable and that "*Virtually no phytoplankton was*

noted in the net hauls on any date, but dark food was found in the guts of copepods collected on June 14 and 18, 1958, and never thereafter." McLaren reported very low 1958 summer primary production values ranging from below detection limits to 59 mg-C/m^2/d (https://wldb.ilec.or.jp/Lake/NAM-32/datalist). Although these earlier methods for estimating primary production would not be currently appropriate for such an ultra-oligotrophic lake, unsuccessful attempts to estimate primary production on Lake Hazen (2012-2017 study) using non-invasive diel O_2 and O_2 stable isotopes, led St. Pierre et al. (20) to conclude that lake productivity remains very low today. GBL has one of the highest recorded transparency for arctic lakes (~30 m) measured by Johnson (9) whereas Rawson (19) reported maximum values of 13-17 m in the deep waters of the East Arm of GSL during the 1940s. Secchi depth measurements on Lake Hazen were taken in 1958 by McLaren (5) where he found highly turbid conditions on before Aug. 9-10 (1.7 m) and then clear water a few days later (after snowmelt) (8.1 m) on Aug. 10-11. These measurements by McLaren were likely taken near their base camp (on the north side of the lake). Although McLaren mentions "open water", it is assumed that this would be near the 1958 base camp, close to northern shore and not near the middle of the lake which is far away from river inputs of glacial flour. In 2014, Secchi depth was measured in the middle of the lake and away from the main input of glacial flour and was found to be 27 m before snowmelt, and 15 m right after snowmelt (20). It is clear that summer turbidity varies spatially across the lake as well as seasonally (before and after snowmelt). Therefore, it is not possible to compare historical and recent Secchi measurements as they were taken at very different locations across this large lake. Nevertheless, since 2007, there has been a large increase in glacial runoff into Lake Hazen with increased turbidity. In the past, the open water period was much shorter and limited to nearshore environments and therefore, a greater proportion of that brief open water period would have been highly turbid.

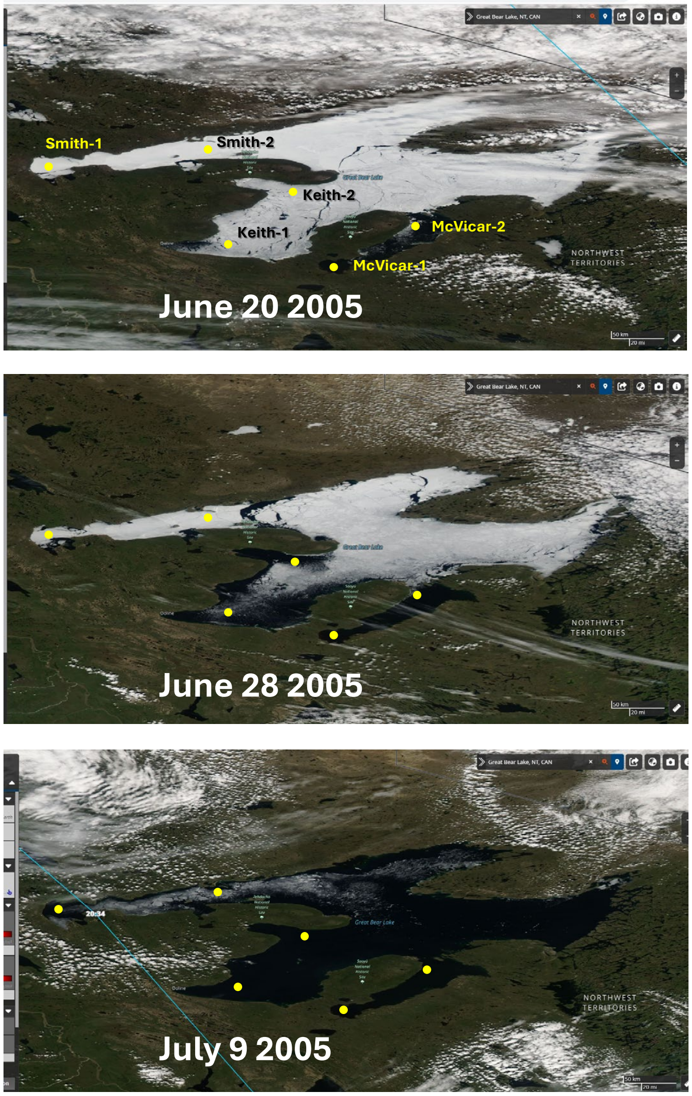

Figure S1: Typical progression of ice-off on Great Bear Lake (GBL). Daily satellite images (NASA MODIS) https://worldview.earthdata.nasa.gov/ taken over GBL in June and July of 2005, showing locations of the six sediment records in this study (yellow circles). These images show the typical progression of ice-off from McVicar Arm in the southeast to Smith Arm in the northwest.

References

1. K.L. Howland *et al*., Combining biological, limnological and palaeolimnological data to study past, present and future impacts of climate change on the Great Bear Lake ecosystem. Northwest Territories Cumulative Impact Monitoring Program (CIMP), Final Report 127 (2020). https://nwtdiscoveryportal.enr.gov.nt.ca/geoportaldocuments/CIMP127%20Final%20Report%20Howland%20et%20al.%20GBL%20July%2013%202020%20with%20Appendices.pdf
2. K.L. Howland *et al*., Combining biological, limnological and palaeolimnological data to study past, present and future impacts of climate change on the Great Bear Lake ecosystem. Northwest Territories Cumulative Impact Monitoring Program (CIMP), Final Report 127 (2020). https://nwtdiscoveryportal.enr.gov.nt.ca/geoportaldocuments/CIMP127%20Final%20Report%20Howland%20et%20al.%20GBL%20July%2013%202020%20with%20Appendices.pdf
3. G. Köck *et al*., Bathymetry and sediment geochemistry of Lake Hazen (Quittinirpaaq National Park, Ellesmere Island, Nunavut). Arctic **65**, 56-66 (2012).
4. I. Lehnherr *et al*., The world's largest High Arctic lake responds rapidly to climate warming. Nat. Comm. **9**, 1290 (2018).
5. I.A. McLaren, Zooplankton of Lake Hazen, Ellesmere Island, and a nearby pond with special reference to the copepod *Cyclops scutifer* Sars. Can. J. Zool. **42**, 613-629 (1964).
6. M.Y. Janjua, R.F. Tallman, K.L. Howland, Elucidation of ecosystem attributes of two Mackenzie great lakes with trophic network analysis. Aquat. Ecosyst. Health Manag. **17**, 151-160 (2014).
7. Sayers et al. 2020
8. A.M. Muir, D.M. Leonard, C.C. Krueger, Past, present and future of fishery management on one of the world's last remaining pristine great lakes: Great Bear Lake, Northwest Territories, Canada. Rev. Fish Biol. Fish. **23**, 293-315 (2013).
9. L. Johnson, Physical and chemical characteristics of Great Bear Lake, Northwest Territories. J. Fish. Board Can. **32**, 1971-1987 (1975a).
10. L. Johnson, The Great Bear Lake: Its place in history. Arctic **28**: 231-244 (1975b).
11. C.E. Herdendorf, Large lakes of the world. J. Great Lakes Res. **8**, 379–412 (1982).
12. Y. Vadeboncoeur, P.B. McIntyre, M.J. Vander Zanden, Borders of biodiversity: life at the edge of the world's large lakes. Bioscience **61**, 526–537 (2011).
13. R.B. Miller, "Great Bear Lake" in *Northwest Canadian fishery surveys in 1944- 45.* (Bull. Fish. Res. Board Canada 72, 1947), pp. 31-44.
14. D.S. Rawson, The net plankton of Great Slave Lake. J. Fish. Res. Board Can. **13**, 53–127 (1956).
15. D.B.O. Saville, General ecology and vascular plants of the Hazen Camp area. Arctic, **17**, 237-255 (1964).
16. E.J. Fee, M.P. Stainton, H.J. Kling, Primary production and related limnological data for some lakes of the Yellowknife, NWT area. Canadian Technical Report of Fisheries and Aquatic Sciences, 1409. Winnipeg, Canada: Fisheries and Oceans Canada (1985).
17. J.W. Moore, Attached and planktonic algal communities in some inshore areas of Great Bear Lake. Can. J. Bot. **58**, 2294-2308 (1980).
18. M.S. Evans, Limnological investigations in the West Basin of Great Slave Lake, March 1994. Northern River Basins Study Project Report No. 131. Edmonton, Alberta: Northern River Basins Study (1997).
19. D.S. Rawson, The physical limnology of Great Slave Lake. J. Fish. Res. Board Can. **8**, 3–66 (1950).
20. K.A. St. Pierre *et al*., Contemporary limnology of the rapidly changing glaciered watershed of the world's largest High Arctic lake. Sci. Rep. **9**, 4447 (2019).

Supplementary Text 2

Scarcity of diatoms and temporal variation in onset of diatom establishment across records

In the earlier sediment intervals of all ten records from GBL, GSL and Lake Hazen, diatoms were too scarce to reliably establish assemblage composition. There was no evidence of dissolution or other preservation issues detected in these earlier intervals, and the rare diatom valves that were encountered were in excellent condition (see ref. 1 for GSL, and ref. 2 for Lake Hazen). It is difficult to conceive how selective dissolution could result in similar trends across three lakes and 10 sedimentary profiles, particularly in GBL where early assemblages consisted of a wide variety of small benthic diatoms as well as large and small cyclotelloid taxa. We discuss this in detail for GSL in the Electronic Supplementary Materials of Rühland et al. (1).

In GBL, variation in the timing of diatom establishment (between ca. 1850 to ca. 1900 in most records, mid-20th century in others; Fig. 1 (main text), Fig. S2) was not unexpected given the lake's vast size and morphological complexity and is likely a product of differences in water column depth, ice cover duration/phenology, nutrient distributions, and other site-specific characteristics (See Table S2 below). For example, the later onset (ca. 1978) at the two deepest sites (McVicar-2 (75 m) and Keith-2 (110 m) that are closer to the central basin where ice cover tends to last longer than the shallower nearshore regions of the arms (Fig. S1 above) may have delayed the establishment of amenable conditions for diatom accumulation in these sediment records. The later onset of diatoms in the temporally shorter record at the nearshore Keith-1 site is more challenging to interpret, but perhaps its location near the source of the Great Bear River (the lake's only outlet), with generally higher wind speeds, stronger circulation and cyclonic gyre patterns in this part of the lake (3) may have affected the sediment depositional environment, delaying diatom accumulation in the sediments of this site.

In GSL, the initiation of diatom establishment in the West Basin also varied across sediment records (Fig. S2). As explained in Rühland et al. (1), the proximity of the coring location to the Slave River and its massive suspended sediment discharge into the West Basin likely delayed the onset of diatom accumulation at these locations. The offshore GSL12 record registered diatoms earlier (ca. mid 19th century) than the GSL19 record (ca. late 1960s) located closer to the Slave River, where highly turbid waters can extend into offshore locations (1) (Fig. 1 main text). Regional warming, reduced precipitation and runoff, together with hydrological regulation with the construction of W.A.C. Bennett Dam in the late 1960s, likely reduced Slave River discharge, changing sediment delivery patterns and increasing summer water clarity, collectively facilitating the accumulation of diatom remains in the sediments at the GSL19 location (1).

Table S2. Physical, chemical and geographical variables for GBL, GSL and Lake Hazen. Physical and chemical limnological data collected at the same locations that sediment cores were retrieved on three arms of Great Bear Lake (March 2018), in the West Basin of Great Slave Lake (March, 1994, March 1995 and March 2014), and from Lake Hazen (no cores taken) July 2003, May/June 2013, 2014, 2017, and August 2015 and 2016.Water samples were retrieved at ~1 m below the water surface for GBl, GSL (through ice), and on Lake Hazen in 2003 in shallow open water. Lake Hazen data from St. Pierre et al. (2019) are depth-integrated samples taken from the deepest part of the lake (~267 m) for 2013, 2014 and 2017 (average of these three years) through ice in May/June, and by boat in August 2015, 2016 (average of these two years). Chemical analyses were undertaken at Environment and Climate Change Canada's National Laboratory for Environmental Testing (NLET) in Burlington, ON for GBL, GSL, and for Lake Hazen in 2003. Chemical analyses were undertaken at the Biological Analytical Service Laboratory (BASL) at the University of Alberta for Lake Hazen data collected from 2013-2017. Water quality data for Lake Hazen collected in 2003 is from Keatley et al. (2007) and data collected from 2013 to 2017 is from St. Pierre et al. (2019).

	Units	GBL Keith-1	GBL Keith-2	GBL McVicar-1	GBL McVicar-2	GBL Smith-1	GBL Smith-2	GSL-13-94	GSL Gros Cap	GSL-12	GSL-19	LH 2003	LH 2015-2016
Date		5-Mar-18	5-Mar-18	8-Mar-18	8-Mar-18	7-Mar-18	7-Mar-18	23-Mar-94	17-Mar-95	30-Mar-14	29-Mar-14	Jul-03	August
Latitude	°N	65.1428	65.6914	64.8769	65.4142	66.1239	66.2336	61.40669	61.885	61.37919	61.41519	81.82283	81.825
Longitude	°W	122.6469	-121.3875	121.2864	120.2636	124.8214	123.7744	115.00028	113.66528	115.35672	114.41389	71.33633	70.7133
Coring Depth	m	45	110	18	75	25	48	62	86	69	43	n.a.	n.a
Ice thickness	cm	78	114	90	105	113	108	137 to 152	n.d.	155	113	n.d.	n.a.
**Field measurements**													
Lake Temperature*	°C	n.d.	n.d	0.0020	0.01	0.02	0.00	0.39	n.d.	0.04	0.07	n.d.	3.230
pH*		n.d.	n.d.	8.14	8.06	8.06	8.21	7.69	n.d.	8.03	8.02	7.73	7.81
Specific Conductivity*	µS/cm	n.d.	n.d.	213	178	198	29.7 [(1)]	240	n.d.	241	239	68	
Dissolved Oxygen *	mg/L	n.d.	n.d.	14.17	14.22	14.49	14.19	13.38	n.d.	13.9	13.9	n.d.	13.6
**Major ions**													
Alkalinity totoal CaCO^3	mg/L	63.1	63.3	85.9	65.5	70.4	64.9	n.d.	n.d.	n.d.	n.d.	n.d.	n.d.
Carbon dissolved inorganic (DIC)	mg/L	13.7	n.d.	18.7	14.1	15.3	13.9	n.d.	n.d.	19.50	19.50	9.0	9.3
Calcium dissolved	mg/L	17.9	17.8	26.1	18.4	20.8	18.2	n.d.	n.d.	30.60	30.60	13.3	n.d.
Chloride dissolved	mg/L	5.4	5.26	7.66	5.56	5.51	5.39	n.d.	n.d.	7.43	7.35	0.17	n.d.
Fluoride dissolved	mg/L	0.08	0.08	0.10	0.08	0.09	0.08	n.d.	n.d.	0.08	0.08	n.d.	n.d.
Magnesium dissolved	mg/L	7.63	7.57	10.4	7.86	8.88	7.78	n.d.	n.d.	7.04	7.05	1.38	n.d.
Potassium dissolved/filtered	mg/L	0.76	0.75	0.88	0.78	0.83	0.79	n.d.	n.d.	1.15	1.16	0.25	n.d.
Silica soluble reactive	mg/L	2.57	2.54	3.02	2.64	2.81	2.61	n.d.	n.d.	n.d.	n.d.	n.d.	0.4
Silica (SiO2)	mg/L	n.d.	n.d.	n.d.	n.d.	n.d.	n.d.	1.50	n.d.	3.95	4.15	0.6	n.d.
Sodium dissolved/filtered	mg/L	4.41	4.31	6.53	4.55	4.69	4.43	n.d.	n.d.	8.23	8.24	0.3	n.d.
Sulphate dissolved	mg/L	15.99	15.87	24.48	16.57	21.08	16.28	n.d.	n.d.	24.50	24.20	6.32	10.5
**Nutrients**													
Chlorophyll A	µg/L	0.40	0.30	0.30	0.20	n.d.	0.30	0.30	n.d.	0.94	0.13	0.05	n.d.
Total phosphorus	µg/L	1.40	1.30	3.60	1.50	2.00	0.80	26	n.d.	6	7	2	2
Phosphorus particulate	µg/L	0.30	n.d.	0.00	n.d.	0.10	0.20	n.d.	n.d.	n.d.	n.d.	n.d.	n.d.
Phosphorus soluble reactive	µg/L	0.10	0.10	0.00	0.00	0.40	0.20	0	n.d.	2	3	n.d.	n.d.
Phosphorus total dissolved	µg/L	0.60	0.70	2.40	0.70	1.00	0.60	11	n.d.	5	5	n.d.	9.5
Ammonia filtered	µg/L	5.00	2.50	2.50	2.50	1.00	4.00	8.00	n.d.	14	10	2.5	n.d.
Nitrite and nitrate filtered	µg/L	177	177	112	179	153	180	96.00	n.d.	10	43	n.d.	n.d.
Nitrogen Kjeldahl total	µg/L	94	89	253	87	135	96	n.d.	n.d.	n.d.	n.d.	40	n.d.
Total nitrogen	µg/L	271	266	365	266	288	276	n.d.	n.d.	n.d.	n.d.	60	60
Nitrogen particulate organic	µg/L	n.d.	n.d.	n.d.	n.d.	n.d.	n.d.	n.d.	n.d.	n.d.	n.d.	16	n.d.
Nitrogen dissolved	µg/L	n.d.	n.d.	n.d.	n.d.	n.d.	n.d.	n.d.	n.d.	273	279	n.d.	n.d.
Nitrogen particulate	µg/L	6.70	3.00	12.20	4.20	7.20	7.20	30	n.d.	57	46	n.d.	8
Carbon dissolved organic (DOC)	mg/L	1.70	n.d.	5.70	1.80	3.00	1.70	5.82	n.d.	n.d.	n.d.	1.1	0.3

*measurements made in the field using a Yellow Springs Instruments (YSI) multiparameter meter at 0.5 m below the water surface

1. Pronounced halocline down to 25 m where specific conductivity reached 162 µS/cm

n.d. = not determined; n.a.= not applicable

GBL = Great Bear Lake; GSL = Great Slave Lake; LH= Lake Hazen

Great Bear Lake full diatom profiles

Smith-1 (coring depth: ~25 m)

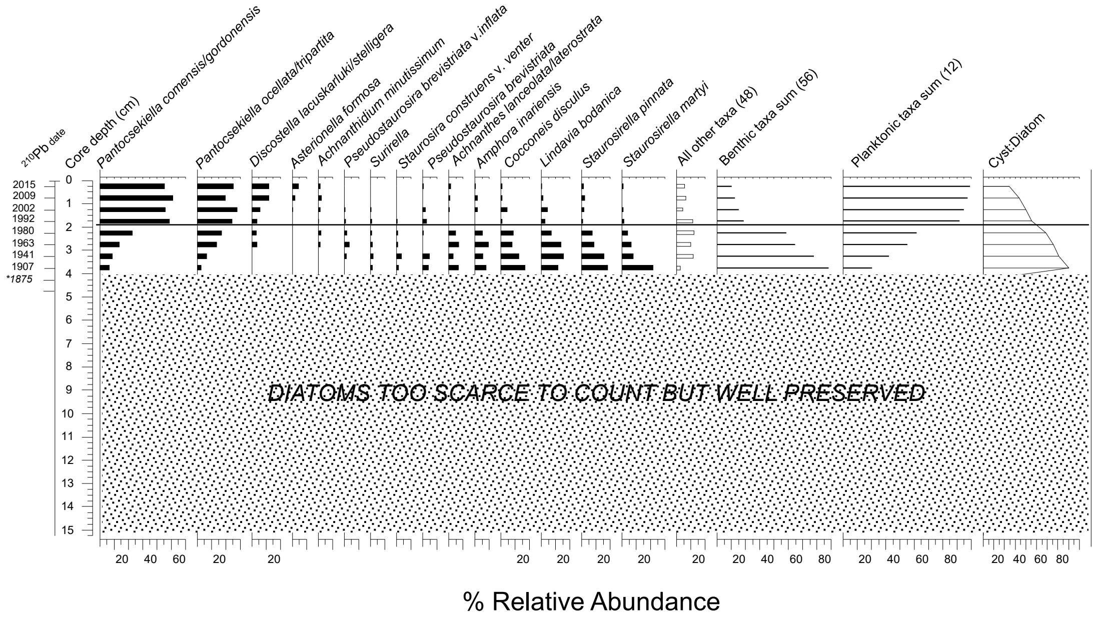

Smith-2 (coring depth: ~48 m)

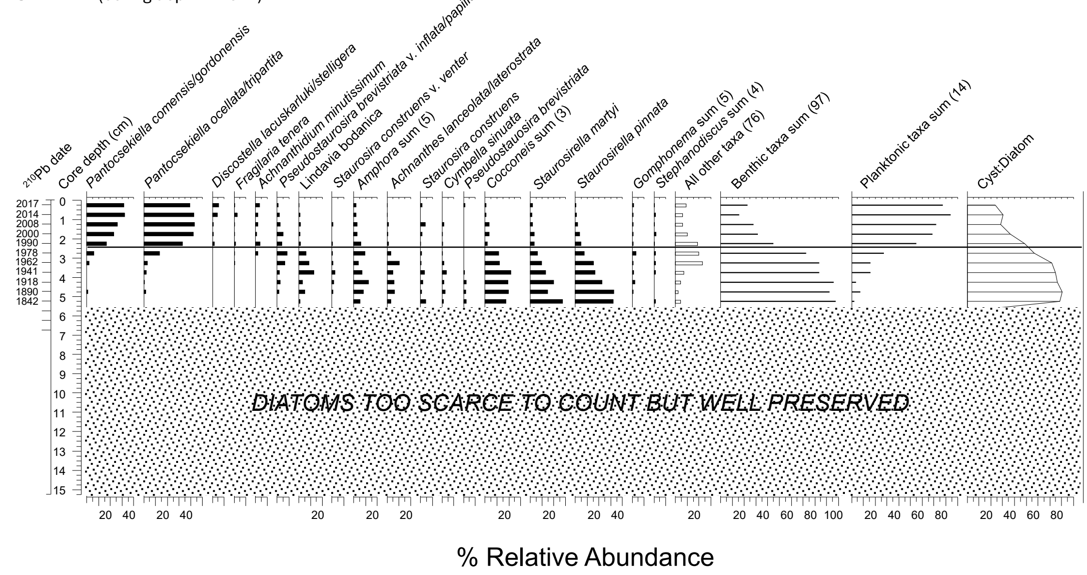

Keith-1 (coring depth: ~45 m)

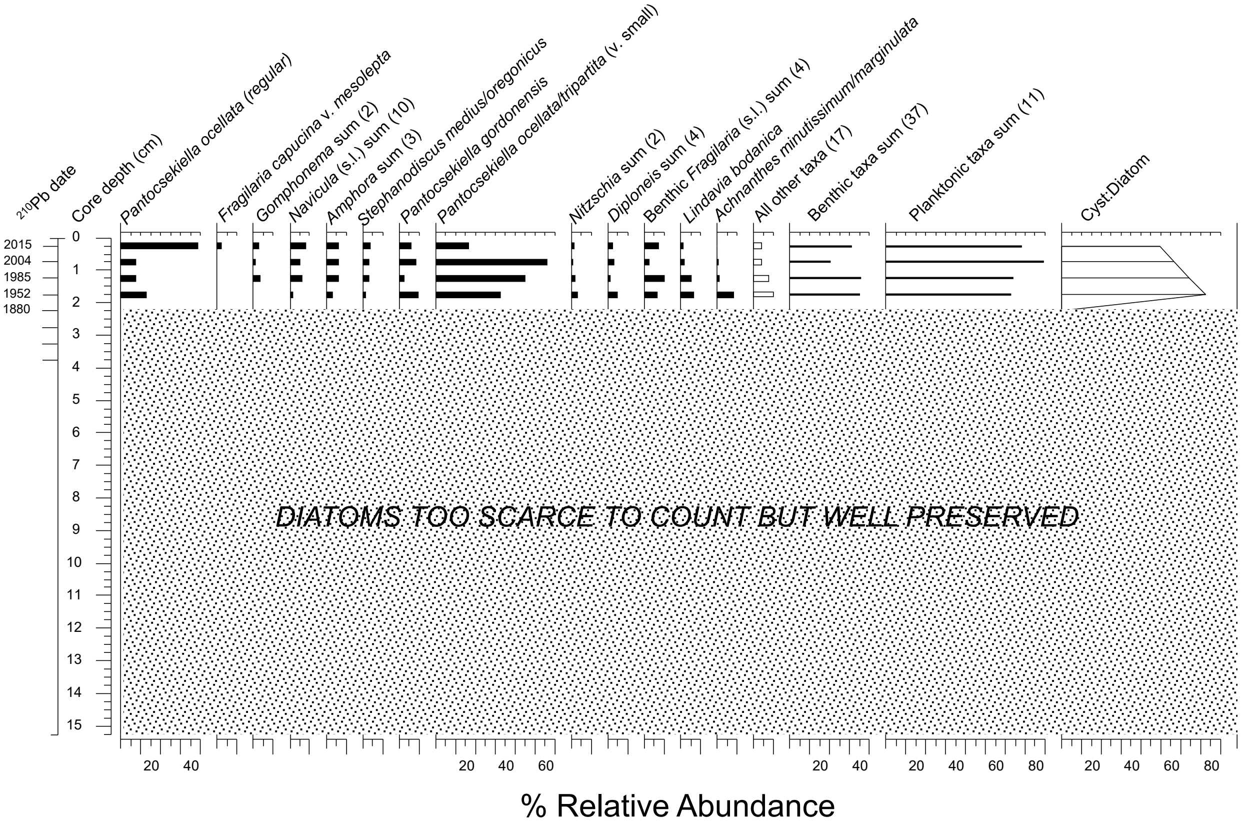

Keith-2 (coring depth: ~110 m)

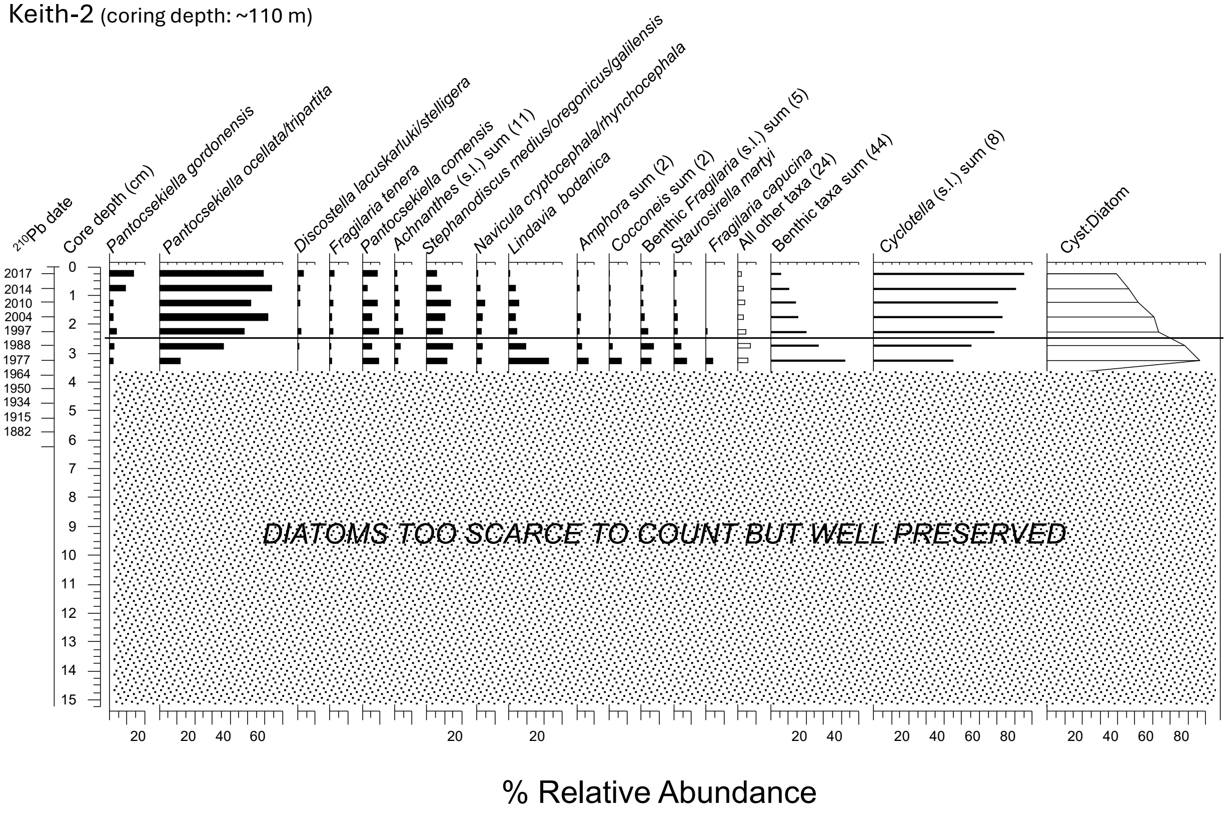

McVicar-1 (coring depth: 18m)

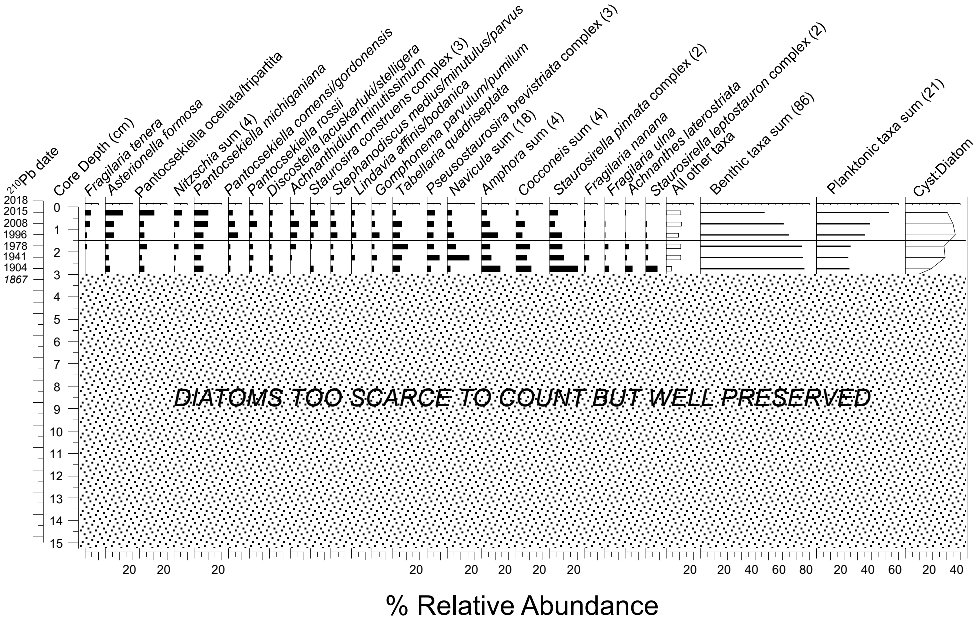

McVicar-2 (coring depth: ~75 m)

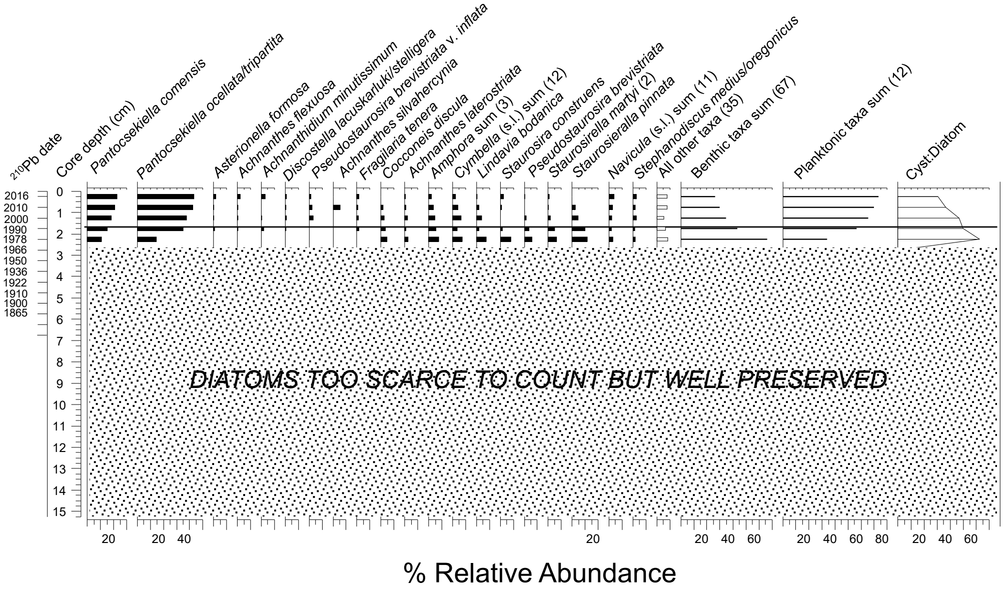

Fig. S2: Great Bear Lake (GBL) Diatom Profiles. The percent relative abundances of the most common diatom taxa observed in the sediment cores retrieved from GBL, NT including from Smith Arm (Smith-1, Smith-2), Keith Arm (Keith-1, Keith-2) and McVicar Arm (McVicar-1, McVicar-2). Numbers in brackets appearing after diatom names indicate the number of taxa that make up that grouping. Diatom biostratigraphical zones (solid vertical lines) were identified by constrained incremental sum of squares (CONISS). Estimated ^{210}Pb dates (based on constant rate of supply (CRS) model) is plotted alongside core depth on the left side of the figure. Also included are the sums of all other taxa that were not included in the figure, all benthic diatoms, all planktonic diatoms, and an index of chrysophyte cysts to diatom frustules (Cyst:Diatom).

References

1. K.M. Rühland, M. Evans, J.P. Smol, Arctic warming drives striking twenty-first century ecosystem shifts in Great Slave Lake (Subarctic Canada), North America's deepest lake. Proc. R. Soc. B 290: 20231252 (2023).
2. N. Michelutti *et al*., Contrasting the ecological effects of decreasing ice cover versus accelerated glacial melt on High Arctic's largest lake. Proc. R. Soc. B **287**, 20201185 (2020).
3. Y. Rao, A. Huang, W.M. Schertzer, W.R. Rouse, Modelling of physical processes and assessment of climate change impacts in Great Bear Lake. Atmosphere-Ocean **50** (3), 317-333 (2012).

Supplementary Text 3

Have Smith, Keith and McVicar arms of GBL historically experienced summer thermal stratification, or is this a new thermal regime?

Our diatom compositional shifts from benthic taxa to greater relative abundances of planktonic *Cyclotella s.l*. taxa registered in GSL, Lake Hazen and the six records from GBL (Smith, Keith and McVicar arms) suggest longer and warmer open water periods with either newly, or more strongly, developed summer thermal stratification since the turn of the 21st century. For GBL, "ground-truthing" our paleo data required gleaning information from rarely reported (often incidental) historical temperature measurements from these three arms: Did they thermally stratify in summer during the early surveys of the 1940s and 1960s, or is this a new thermal regime? Below, we collect supporting evidence gleaned from historical surveys of GBL to support our paleolimnological conclusions that this is indeed a new thermal regime.

Did the protected arms of GBL historically experience summer thermal stratification?

Data collected in July and August during the 1960s (1, 2) focused on McTavish Bay (6 miles from Port Radium, near deepest part of GBL) and showed isothermal temperature profiles (~4°C). At approximately the same location as Johnson's surveys, recent surface temperature data (3) collected during the open water seasons since 2000 and water column temperature profiles since 2012 (Fisheries and Oceans Canada (DFO) in partnership with the Délı̨nę, Renewable Resources Council (DRRC) and the Sahtu Renewable Resources Council (SRRB)) show an increase in surface water temperatures (3) and the development of summer thermal stratification in most years, with the exception of occasional deeper, offshore regions of the bay that in some years, remained isothermal (4).

However, have the more protected arms of GBL (Smith, Keith, McVicar) where our sediment cores were collected, also undergone a change in response to longer and warmer open water periods and either newly, or more strongly, developed summer thermal stratification, consistent with our diatom compositional changes? The scarcity of historical temperature readings in these parts of the lake make it challenging to "ground-truth" whether summer thermal stratification is a new development (more persistent and stronger), or if they have historically experienced summer thermal stratification. Below we look at historical reports to glean answers to these questions.

The 1945 summer survey of GBL by Miller and Kennedy described the lake in great detail (5–7), but surface water temperature or water column profiles they measured were often mentioned only incidentally – especially in the more protected arms of the lake. Miller noted that surface water

temperatures in the protected arms were warmer than in the open lake but below the surface, the water remained uniformly cold. Kennedy (5) and Miller and Kennedy (6) provide some evidence that during the summer of 1945 the southern arms were oligotrophic and (with a few exceptions like the tip of McVicar Arm) likely did not thermally stratify (or at least not strongly). During the 1940s, Miller and Kennedy set up camp on Keith Arm near Délı̨nę (then Fort Franklin) where they focused on Miller's fisheries study, but they also made trips to Smith and McVicar arms to study whitefish fisheries. They reported that the average temperature Délı̨nę was slightly higher than that of the east end of McTavish Arm (deeper and colder part of lake), although exact locations and actual temperatures were not specified. However, Miller (6) mentioned that on the east shore near Port Radium (this may be closer to shore than where Johnson later took temperature profiles), the surface temperatures at the end of July was 43.2 °F (6.2 °C). Miller and Kennedy (7) also note that many of the bays they sampled within Keith, Smith and McVicar arms were sufficiently isolated from the open lake and water temperatures were notably higher. In Conjuror Bay (inland secluded bay near Port Radium), Miller (6) mentions that surface waters reached as high as 63.1 °F (17.3 °C) and the field party indulged in its only bath. Miller and Kennedy (7) describe one bay as being anomalous in terms of the higher amount of lake trout caught at depth (they stress that this was not so in any other bays) and attributed this anomaly to pronounced thermal stratification at this one site. Unfortunately, they do not indicate which bay this was, other than it was a "non-marshy" bay, relatively shallow and sufficiently isolated from the open lake that temperatures were appreciably higher. These reports suggest that, with the exception of a few of the most isolated and shallow bays, most parts of GBL that Miller and Kennedy explored during the 1945 survey, did not experience summer thermal stratification (or it was weakly developed).

Moore (8) focused on nearer to shore parts of the lake including a few temperature recordings on Keith Arm near Fort Franklin (now Délı̨nę) in 1976. Although the information is scant and continuous temperature recordings with depth were not measured, Moore notes that the sampling sites near Fort Franklin were ice covered in July 1976 with temperatures ranging from 0 °C to 1 °C, increasing during August to 6 °C to 7 °C at the surface and 4 °C at 30 m. By September, the water cooled slightly to 4 °C to 4.5 °C, regardless of depth. This suggests that summer thermal stratification in 1976 was weak, at best, in Keith Arm. In comparison, recent water temperatures measured during August at Keith Arm (3, 4, 9, 10) show warmer temperatures than historical data. For example, mean August surface water temperatures (2000-2003, 2005, 2006, 2007, 2012, 2014, 2015, 2017, 2018, 2021, 2022, 2023) exceeded 10°C in most years, even at the deepest locations of Keith Arm (3). In addition, summer thermal stratification developed near Délı̨nę in August 2008 (10) and at some point in all years measures and at

all sites (4, 9). In Smith Arm located in the more northwesterly region of GBL, mean July and August surface water temperatures (measured in 2006, 2011, 2016, 2022, 2023) were rarely below 10°C (3), whereas water column temperature profiles indicated that summer thermal stratification developed in all years with the exception of one deeper offshore station in 2016 that recorded isothermal conditions (4). In agreement with our paleolimnological changes, warmer surface water temperatures (3) and recently measured temperature profiles (4, 9, 10) indicate that in contrast to historical records, these three arms of GBL commonly and clearly thermally stratify for extended periods during the increasingly common warmer summers.

The circular tip of McVicar Arm (where our McVicar-1 core was collected at ~18 m) receives warm water from the Johnny Hoe River and likely experienced periods of thermal stratification during the 1960s (2) and perhaps also during Miller's 1940s survey. Johnson (11) described it as the largest mass of warm water within the whole lake, with 1964 summer surface temperatures at the center reaching 13 °C with a bottom temperature (at ~35 m, maximum depth of this basin) of 11 °C, therefore only a 2 °C difference (i.e., weakly stratified). Although water column temperature profiles were not reported, August surface water temperatures measured by Johnson in the shallowest areas reached 14.5 °C to 15 °C in 1963 and 1964 (Fig. S3). Johnson regarded this basin as separate from the rest of GBL and classified it as dimictic (11). More recently, mean July and August surface water temperatures measured at this location in 2013 and 2018 exceeded 10°C with some sites in 2018 exceeding 16°C (3). Temperature profiles collected at McVicar Arm in August 2013 and 2018 showed a thermally stratified water column during July and August at all stations and at all depths (4, 9). Although this most southern part of GBL likely historically experienced periods of thermal stratification during the summer, for at least the past ~60 years, the increase in planktonic cyclotelloid relative abundances in recent decades registered in our diatom records (Fig. 1 of main paper), together with recent water temperature measurement, suggests that thermal stratification is currently more pronounced and likely more prolonged than in the past.

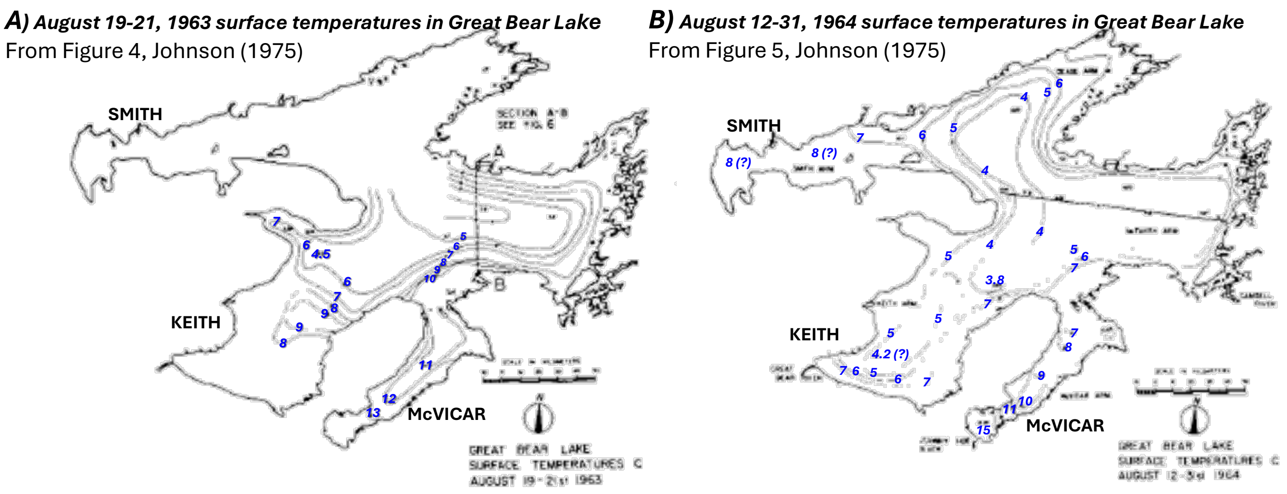

Fig. S3. Distribution of surface water temperatures measured in Great Bear Lake in A) August 19-21, 1963 and B) August 12-31, 1964, modified from Figures 4 and 5 in Johnson (2). Most of Keith Arm was not much higher than 4 °C (especially in 1964). In McVicar Arm, surface temperatures were highest at the most southern part, especially in the circular basin where temperatures reached a little over 13 °C in both years. The Johnny Hoe River discharges warm waters at this temperature in midsummer (2). Johnson (2) noted that as one proceeded north up McVicar arm, surface temperatures decreased in a regular manner until the region of deepest water was reached and the lowest surface temperatures of the arm were recorded (A and B). However, there was a considerable difference between A) 1963 (11 °C) and B) 1964 (7 °C). Where our core McVicar-2 (~75 m) was collected, surface temperature in 1964 was ~6 °C. Today, surface temperature at this location was ~16 °C in 2018) and summer thermal stratification and thermocline are well developed (4, 9).

References

1. L. Johnson, Temperature of maximum density of fresh water and its effect on circulation in Great Bear Lake. J. Fish Res Bd Canada **23**, 963-973 (1966).
2. L. Johnson, Physical and chemical characteristics of Great Bear Lake, Northwest Territories. J. Fish. Board Can. **32**, 1971-1987 (1975).
3. DFO (Fisheries and Oceans Canada) / Pêches et Océans Canada (MPO), Data from "Great Bear Lake (Sahtú) Surface water temperature monitoring" (dataset). 1.0.0. DataStream. https://doi.org/10.25976/2u81-ra56. Deposited 2025.
4. K.L. Howland, et al., Monitoring for impacts of harvest and climate change on the Great Bear Lake aquatic system. Northwest Territories Cumulative Impact Monitoring Program (CIMP), Final Report (2021-23) 127 (2024). https://nwtdiscoveryportal.enr.gov.nt.ca/geoportaldocuments/2023-24%20-%20Final%20Report%20-%20DFO(Howland)%20-%20CIMP127%20-%20Sep%2017%2C%202024.pdf
5. W.A. Kennedy, The Coregonine fish of Great Bear Lake, N.W.T. Bull. Fish. Res. Board Can. No. 483, 40 pp. (1947).
6. R.B. Miller, "Great Bear Lake" in *Northwest Canadian fishery surveys in 1944- 45.* (Bull. Fish. Res. Board Canada 72, 1947), pp. 31-44.
7. R.G. Miller, W.A. Kennedy, Observations on Lake Trout of Great Bear Lake. J. Fish. Res. Bd. Canada **7**, 176-189 (1948).
8. J.W. Moore, Attached and planktonic algal communities in some inshore areas of Great Bear Lake. Can. J. Bot. **58**, 2294-2308 (1980).
9. K.L. Howland, *et al*., Combining biological, limnological and palaeolimnological data to study past, present and future impacts of climate change on the Great Bear Lake ecosystem. Northwest Territories Cumulative Impact Monitoring Program (CIMP), Final Report 127 (2020). https://nwtdiscoveryportal.enr.gov.nt.ca/geoportaldocuments/CIMP127%20Final%20Report%20Howland%20et%20al.%20GBL%20July%2013%202020%20with%20Appendices.pdf
10. E. Carmack, S. Vagle, H. Kheyrollah Pour, Seasonal temperature and circulation patterns in a hybrid polar lake, Great Bear Lake, Canada. J. Geophys. Res.: Earth Surf. **129**, e2024JF007650 (2024).
11. L. Johnson, Distribution of fish species in Great Bear Lake, Northwest Territories, with reference to zooplankton, benthic invertebrates, and environmental conditions. J. Fish. Res. Board Can. 32: 1989-2004 (1975).

Supplementary Text 4

Implications of climate change and phytoplankton compositional shifts on fishes

Longer and warmer open-water periods and increased summer thermal stability can reduce the preferred oxythermal habitats for many cold-adapted fish species such as Lake Trout (*Salvelinus namaycush*), one of the top predators in GBL and GSL, that has a narrow tolerance range (<12 to 15° C; Ref. #1). As discussed in the main text and in Supplementary S3, water temperatures have already exceeded this narrow range in some arms of GBL. Preliminary trends in historical and recent biological data suggest that Lake Trout catch composition and size have remained relatively stable across the lake, although at some locations there is evidence of temporal variation in mean size (2). Trophic modelling of GBL and GSL indicates that these ecosystems are currently stable (3) but continued warming and associated shifts in diatom assemblages is expected to impact the distribution of key fish species and energy flow through the system which is heavily dependent on phytoplankton at the base of the food web (4, 5). On Lake Hazen, there is evidence that, over the past 25 years, Arctic Char (*Salvelinus alpinus*) are being affected by climate-related increases in glacial runoff and minerogenic turbidity that are impacting the feeding tactics of this visual predator, with significant declines in their physiological condition (6). Since the 1990s, declines in mercury concentrations in large and small char morphotypes were associated with increased glacial runoff and increased snow, respectively (7). Interestingly, 25 years of $\delta^{13}C$ data on muscle samples indicate that both small (historically bottom feeders) and large (piscivorous) morphs of Lake Hazen Arctic Char are moving toward a more pelagic influenced diet (8; D. Muir; pers. comm).

As warming is predicted to continue, it would be ideal to continue year-round monitoring and sampling of water quality parameters together with phytoplankton and zooplankton collections. The shift from large (lipid-rich) *Aulacoseira* taxa to fast-growing cyclotelloid taxa in GSL, requires more information before it can be determined how this will impact upper trophic levels. However, similar scenarios to GSL were reported in the more southern LGLs (9) where the impacts of a shift to smaller cyclotelloid taxa on food web dynamics were well documented (10). For example, by examining diatom species composition in a sediment record and from the gut content of historical collections of benthic amphipods (*Diporeia*) from southern Lake Michigan, Edlund et al. (10) were able to determine that the loss of this critical benthic-pelagic link, that had previously accounted for up to 65% of benthic biomass in Lake Michigan (11), was associated with a significant shift in the quality and size of its food source. Similar to our GSL sediment records, large and calorie-rich *Aulacoseira* and *Stephanodiscus* taxa

dominated earlier diatom assemblages during the spring bloom, providing *Diporeia* with a lipid-rich diet and, in turn, served as the primary and preferred prey for many deepwater and nearshore fish species. However, at the turn of the 21st century, an increase in abundance of nutrient-poor, small-celled cyclotelloid taxa was determined to have played a major role in the functional extinction of *Diporeia* in Lake Michigan (and other LGLs) that altered the energy flow throughout the entire food chain of this large freshwater system (10). These direct linkages using species-specific analysis between trophic levels provides support that a shift in phytoplankton composition can lead to food limitation with repercussions throughout the food web. Even in the LGL, which have been much more intensively studied than GSL, GBL and Lake Hazen combined, there remains much to understand about the environmental consequences to food web dynamics (9).

References

1. M.M. Guzzo, P.J. Blanchfield, Climate change alters the quantity and phenology of habitat for lake trout (Salvelinus namaycush) in small Boreal Shield lakes. Can. J. Fish. Aquat. Sci. **74**(6), 871–884 (2017).
2. K.L. Howland, et al., Monitoring for impacts of harvest and climate change on the Great Bear Lake aquatic system. Northwest Territories Cumulative Impact Monitoring Program (CIMP), Final Report (2021-23) 127 (2024). https://nwtdiscoveryportal.enr.gov.nt.ca/geoportaldocuments/2023-24%20-%20Final%20Report%20-%20DFO(Howland)%20-%20CIMP127%20-%20Sep%2017%2C%202024.pdf
3. M.Y. Janjua, R.F. Tallman, K.L. Howland, Elucidation of ecosystem attributes of two Mackenzie great lakes with trophic network analysis. Aquat. Ecosyst. Health Manag. **17**, 151-160 (2014).
4. L. Chavarie, K. Howland, C. Gallagher, C. and W. Tonn, W. Fatty acid signatures and stomach contents of four sympatric Lake Trout: assessment of trophic patterns among morphotypes in Great Bear Lake. Ecol. Freshw. Fish **25**: 109-124 (2014).
5. K.L. Howland, *et al.*, Combining biological, limnological and palaeolimnological data to study past, present and future impacts of climate change on the Great Bear Lake ecosystem. Northwest Territories Cumulative Impact Monitoring Program (CIMP), Final Report 127 (2020). https://nwtdiscoveryportal.enr.gov.nt.ca/geoportaldocuments/CIMP127%20Final%20Report%20Howland%20et%20al.%20GBL%20July%2013%202020%20with%20Appendices.pdf
6. I. Lehnherr *et al.*, The world's largest High Arctic lake responds rapidly to climate warming. Nat. Comm. **9**, 1290 (2018).
7. K. Hudelson *et al.*, Mercury at the top of the world: A 31-year record of mercury in Arctic char in the largest High Arctic lake, linked to atmospheric mercury concentrations and climate oscillations. Env. Pollut. **337**, 1224566 (2023).
8. A. Cabrerizo, D.C.G. Muir, G. Köck, D. Iqaluk, X. Wang, Climatic influence on temporal trends of polychlorinated biphenyls and organochlorine pesticides in landlocked char from lakes in the Canadian High Arctic. Environ. Sci. Technol. **52**: 10380-10390 (2018).
9. E.D. Reavie *et al.*, Climate warming and changes in *Cyclotella sensu lato* in the Laurentian Great Lakes. Limnol. Oceanogr. **62**, 768–783 (2017).
10. M.B. Edlund, D.J. Jude, T.F. Nalepa, Diets of the benthic amphipod Diporeia in southern Lake Michigan before and after the dreissenid invasion. J. Great Lakes Res. **47**, 447-462 (2021).
11. T.F. Nalepa, Estimates of macroinvertebrate biomass in Lake Michigan. J. Great Lakes Res. **15**, 437-443 (1989).

Supplementary Methods

<u>Expanded Methods and Results</u>

Sediment coring and water chemistry:

GBL: Sediment cores were retrieved through ice in March 2018 from six locations (Fig. S1) in three arms of GBL (McVicar, Keith, Smith arms) using a UWITEC gravity corer. Coring locations and depth of retrieval include McVicar-1 (68.8769°N, 121.2864°W) at ~18 m, McVicar-2 (65.4142°N, 120.2636°W) at ~75 m, Keith-1 (65.1428°N, 122.6469°W) at ~45 m, Keith-2 (65.6914°N, 121.3875°W) at ~110 m, Smith-1 (66.1239°N, 124.8214°W) at ~25 m, and Smith-2 (66.2336°N, 123.7744°W) at ~48 m. These locations were selected to provide a spatially extensive representation of this large lake and its various river influences (e.g., the Johnny Hoe and Whitefish rivers flow into McVicar and Smith arms, respectively, whereas Keith Arm is the location of the lake's only major outflow- into the Great Bear River).

The six GBL cores were sectioned on site into 0.5 cm intervals for the first 20 cm of the core, then into 1 cm intervals for the remainder of the cores. The sediment sections were placed into plastic Whirl-Pak® bags and kept cool before being transported to ECCC in Saskatoon where the samples were subsequently freeze-dried before being shipped to Paleoecological Environmental Assessment and Research Lab (PEARL), Queen's University, Kingston, Ontario for analyses.

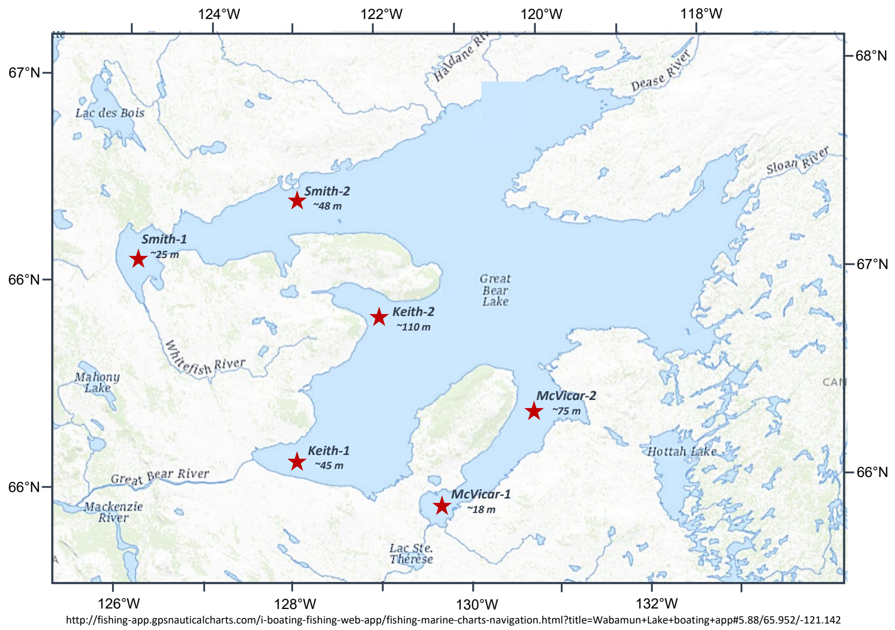

Fig. S4: Location of 2018 coring sites (red stars) on Great Bear Lake (N.W.T., Canada) and corresponding coring depth.

<u>GSL and Lake Hazen</u>: Two sediment cores were retrieved from the West Basin by ECCC using a UWITEC gravity corer including GSL12 (61.37919°N, 115.35672°W: ~69 m lake depth) and GSL19 (61.41510°N, 114.41389°W: ~47 m lake depth). Sediment cores from two locations on Lake Hazen were collected using a UWITEC gravity corer including from the Blister site (81.79171°N, 71.46929°W: ~258 m lake depth) in 2017 and from the main basin site (81.82493°N, 70.71498°W: ~260 m lake depth) in 2013. Sediment sampling details for GSL can be found in Rühland et al. (1) and for Lake Hazen in Michelutti et al. (2).

Radiometric dating:

Approximately 20 strategically selected sediment intervals for each of the six cores retrieved from GBL, the two cores from GSL, and the two cores from Lake Hazen were analysed by gamma spectrometry at PEARL, Queen's University, Canada. Prior to ^{210}Pb analysis, sediment samples were freeze dried at ECCC laboratories in Saskatoon (GBL, GSL) or at PEARL (Lake Hazen), noting the dry weight and percent moisture of each sample. Following Schelske et al. (3), approximately 1.5 grams of freeze-dried sediment were placed into plastic gamma tubes and sealed with 2-ton epoxy. For each prepared sample, activities of ^{210}Pb, ^{137}Cs, and ^{214}Pb (proxy for supported/background ^{210}Pb) were measured with a well-type, Ortec high-purity Germanium detector. The chronologies for all lakes were established based on estimates of ^{210}Pb activities using the constant rate of supply (CRS) model (4) and the program ScienTissiME (<u>http://www.scientissime.net/software)</u>. ^{137}Cs was considered as a potential independent chronological marker of the peak in radioactive fallout resulting from the 1963 global moratorium on nuclear weapons testing.

The dating profiles for each of the six cores studied in GBL (Smith, Keith, and McVicar arms) are shown in Fig. S5. Initial total ^{210}Pb concentrations for the six GBL cores ranged from 449.8 Bq/kg (McVicar-1) to 1977.6 Bq/kg (Smith-1) and generally followed an exponential decline with core depth. With a few exceptions, ^{137}Cs showed peak values in most cores that generally corresponded to with the1963 height of nuclear weapons testing, particularly given the relatively coarse temporal resolution (i.e., the interval containing the peak activities includes 1963 but could represent numerous years). McVicar-1, 2.25 cm = 1941 ±5.5; McVicar-2, 2.75 cm = 1966 ±3.5; Keith-2, 3.25 cm = 1977 ±1.5; Smith-1, 2.75 cm = 1963± 3.1(but topmost sample (2015) broke the trend with highest value in core); Smith-2, 3.25 cm = 1962 ±2.0. The ^{137}Cs profile for Keith-1 had an increasing trend towards top of the core, suggesting post depositional ^{137}Cs migration. Comparison of sediment accumulation rates among GBL cores (also with GSL coring sites) are shown in Fig. S6.

Dating results for GSL and Lake Hazen can be found in the electronic supplementary material, Rühland et al. (1) and Michelutti et al. (2), respectively.

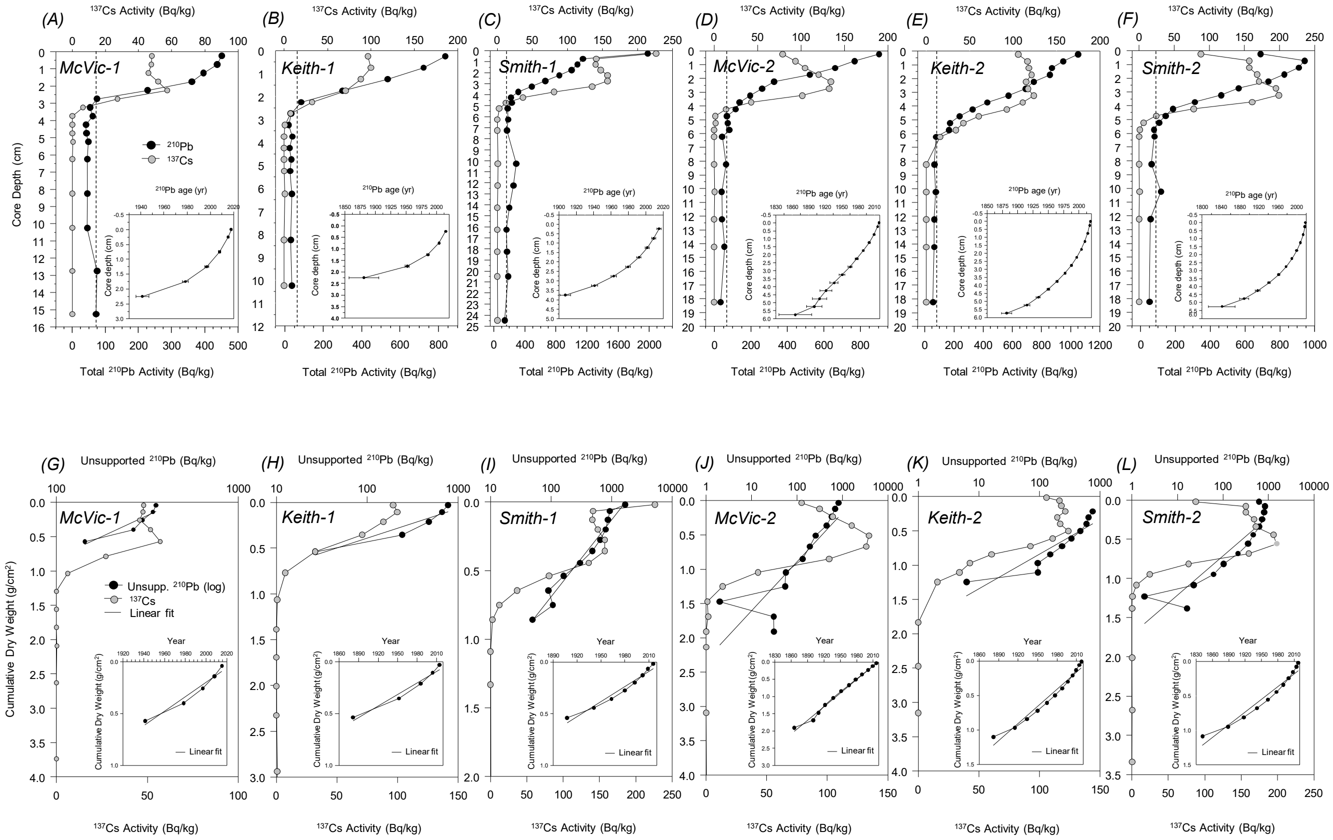

Fig. S5. Radiometric dating analysis for Great Bear Lake sediment cores. A-F: Total ^{210}Pb (black circles) and ^{137}Cs (grey circles) activities plotted against core depth for (A) MicVicar-1, (B) Keith-1, (C) Smith-1, (D) McVicar-2, (E) Keith-2, and (F) Smith-2. The vertical dashed lines represent the mean of supported ^{210}Pb (background) levels using ^{214}Pb as a proxy. The age-depth models (insets) are included for each core and includes associated error bars (accumulated uncertainties in the estimated age model). Dates were based on the constant rate of supply model (CRS). G-L: The log unsupported ^{210}Pb activities plotted against cumulative dry weight (and linear fit lines) for (G) MicVicar-1, (H) Keith-1, (I) Smith-1, (J) McVicar-2, (K) Keith-2, and (L) Smith-2. Insets depict estimated age versus cumulative dry weight profiles with linear fit lines. All cores were collected by ECCC in 2018 and dated at PEARL.

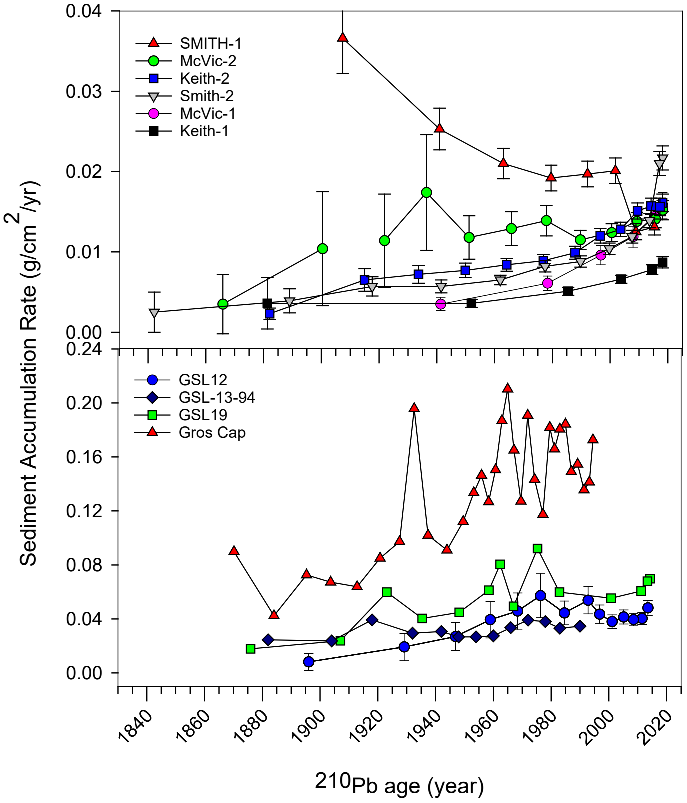

Figure S6. Sediment accumulation rates based on ^{210}Pb dating analysis: Six GBL sites and four GSL sites. Note the generally lower rates in GBL compared to GSL. GSL sites with higher sediment accumulation rates (GSL19, Gros Cap) were located closest to the Slave River (1).

Diatom analysis: Diatom sample preparation for all cores followed PEARL methods, outlined in Rühland and Smol (5). Approximately ~0.02 to 0.05 grams of freeze-dried (or ~0.2 to 0.5 grams of wet) sediment were placed into 15 ml glass scintillation vials and then treated with a 50:50 molar mixture of nitric and sulfuric acids to digest the organic sediment matrix. The vials were placed into a hot water bath set to 80°C for at least one hour before being removed and allowed to settle for approximately 24 hours. The supernatant was removed and then samples were rinsed with deionized water and diatoms allowed to settle: this procedure was repeated until a litmus test indicated samples were no longer acidic. The resulting diatom slurries were “decanted” prior to plating to eliminate the majority of heavier siliciclastic material (particularly for GSL). Small aliquots of the resulting slurries were strewn onto coverslips in different dilutions and allowed to evaporate before being permanently fixed onto microscope slides with Naphrax®, a high refractive index mounting medium. For all cores, diatoms were identified and enumerated using oil immersion lenses on Leica DMRB microscopes (1000 x magnification) with differential interference contrast optics. Diatom taxonomy was established through a variety of sources (6-10). Current taxonomic names were updated from Algaebase (https://www.algaebase.org/Guiry and Guiry). Where possible (i.e. in intervals where diatoms were relatively plentiful), 300-350 diatom valves were counted. In the deeper sediment intervals of all cores, diatom enumeration became challenging and required substantially increased counting effort to attain ~100 valves (approximately the entire cover slip). In intervals where silicilastic materials were high and diatoms were scarce, the glass vials of diatom slurries were shaken and then quickly ‘decanted’ into a clean vial, leaving behind the heavier grains. The resulting diatom slurry was then allowed to settle for 24 hours before removing most of the supernatant (overlying distilled water) from the vial. These steps allowed for a cleaner and more concentrated diatom slide (magnitudes higher than a typical diatom sample). In several GSL samples (lower intervals) where this approach did not yield satisfactory results (i.e. the siliciclastic material was not heavy enough for decanting), a density gradient separation technique using sodium polytungstate (SPT) was applied to remove excessive siliciclastic material and to concentrate diatom valves on the microscope slides (Tapia and Harwood, 2002; see details in the Supplementary Materials of Rühland et al. 2023). The entire sample of highly concentrated slurries (again, magnitudes higher than a typical diatom sample) were strewn onto two coverslips and allowed to evaporate before being mounted onto microscope slides with Naphrax® and enumerated (if possible). For some of these deeper sediments where diatoms were less plentiful, at least ~100 valves were typically counted except for four intervals where diatoms were so sparse that only ~ 80 valves were counted (even after covering most of the coverslip). Counting effort was deemed impractical when less than 10 valves (albeit very well preserved)) were encountered in

~200 fields of view of the concentrated coverslips. Although one could count a few thousand fields of view to obtain a count of 100, we would argue that this degree of diatom scarcity tends to result from "survivors" and would therefore, not be strong indicators of environmental conditions.

Diatom data were presented as percent relative abundances. To facilitate comparisons across diatom profiles and with instrumental records, the main taxa (*Cyclotella sensu lato* species (GBL, GSL, Lake Hazen), benthic sum (GBL), benthic fragilarioid taxa (Lake Hazen) and *Aulacoseira islandica* (GSL) were presented as Z-scores (standardized within cores to a mean of 0 and a standard deviation of 1).

Spectral analysis: Visible range spectroscopy-inferred chlorophyll a (VRS-Chla):

Visible range spectroscopy-inferred chlorophyll a (VRS-Chla) include the main diagenetic products of chlorophyll-*a* and all of its isomers (11, 12). For each sample analyzed, a small amount of freeze-dried sediment was sieved using a mesh size <125 µm to equalize sediment grain size and the influence of water. Sediment spectra were obtained using a FOSS NIRSystems Model 6500 series Rapid Content Analyzer, operating over the range of 400-2500 nm. Trends in sediment chlorophyll-*a* concentrations were inferred using a log-transformation of the published algorithm (11). Lake Hazen VRS-Chla results were too low to report.

Our sedimentary VRS-Chla trends for a subset of the GBL profiles (Smith-2, Keith-2, McVicar-2) and GSL (GSL12, GSL19) were compared to the Sayers et al. (13) primary productivity data reported for these two lakes and derived from remote sensing between 2003 and 2018 (available for download as part of Sayers et al. (13) supplementary materials). From Sayers et al. (13), the remote sensing data represent the photic zone averages for the ice-free period of each year. Remotely sensed imagery were collected for each lake between January 1, 2003 and December 31, 2018. Due to extended periods of ice and cloud cover, annual mean production maps for GSL and GBL were calculated from the months of June to November. See Sayers et al. (13) for greater detail on satellite-derived primary production data. To facilitate comparisons between the remote sensing data (13) and the paleo datasets, all data were converted to Z-scores and linear trendlines were compared over a similar time period, acknowledging that paleo data have coarser temporal resolution than the annually resolved remote sensing data. Mann-Kendall tests were applied to determine if the trends were significantly monotonic. VRS-Chla trends for all records in GBL and GSL showed significant increasing monotonic trends (McVicar-2: $R^2 = 0.90$, $p<0.05$; Keith-2: $R^2=0.91$, $p<0.003$; Smith-2: $R^2 = 0.90$, $p<0.02$; GSL19: $R^2 = 0.96$, $p<0.0003$; GSL12: $R^2 = 0.81$, $p<0.002$) that are consistent with the significant increasing trends in primary production over this same time period for these two lakes reported by Sayers et al. (13) (GBL: $R^2 = 0.35$, $p=0.16$; GSL: $R^2 = 0.30$, $p= 0.028$).

Climate and instrumental data

Weather station records:

Selection of representative weather station data: Historical trends in air temperature and wind speed using data obtained from all available nearby weather stations were scrutinized to select the longest, continuous record that is most representative of the region where the sediment cores were collected (Table S3). Based on comparisons and assessments of mean annual air temperature (MAAT) and wind speed data from all available weather stations in proximity to each lake (from Environment and Climate Change Canada and Parks Canada), it was determined that the longest and most continuous record that best represented our lake locations was Norman Wells (GBL): temperature data (1944-2019), station 2202810; wind speed data (1944-2017), station 2202800; Hay River (GSL): temperature data (1896-2019), station 2202402; wind speed data (1953-2014, station 2201700); and Eureka (L. Hazen): temperature data (1948-2019), station 2401199; wind speed data (1953-2014), station 2401200. To facilitate comparisons between instrumental records and paleo data, all data sets covered similar time periods between 1948 to 2019. For all stations, mean annual air temperature (MAAT) and mean annual wind speed data were presented as anomalies data calculated from the same 30-year period (1981-2010 mean). The details of these assessments are provided below.

Air temperature and particularly wind speed at both Alert and Eureka climate stations are well known to differ from conditions at Lake Hazen with its inland location near mountains contributing to the Hazen basin having an unusual wind regime (14). Although weather stations supported by Parks Canada established on Lake Hazen itself (1988-2009) and at Tanquary Fiord airport (from 1989 and currently still recording) would be ideal choices, these records are short and contained many missing data. Lake Hazen is approximately 150 km from the Alert station and ~300 km from the Eureka station, and based on distance, it would seem reasonable to select the Alert station to represent the Lake Hazen region. Although both stations differ to the Hazen basin region (14), the very coastal location of Alert was found to be differ more compared to the inland location of Lake Hazen (Table S3)- which is considered to be an Arctic oasis in a rain shadow. Although MAAT data from Eureka (1947-2019) and Alert (Environment Canada station id 2400305, temperature data available between 1951-2019) stations were highly correlated ($R = 0.85$, $p<0.00001$), wind speed data (1953-2014) differed considerably between these stations (Table S3, $R= 0.22$, $p = 0.09$), particularly after ca. 1990, where they displayed opposite trends (likely due to Alert's coastal location) (Fig. S7). To assess the best match for Lake Hazen temperature and wind, we made comparisons to data available (requested with permission from Parks Canada) from two weather stations supported by Parks Canada including a station located on Lake Hazen

(closed in 2009) and at the Tanquary Fiord Airport (currently still recording) that is close to Lake Hazen. Although short, historical wind data from Tanquary Fiord Airport (Parks Canada, with permission available between 1989-2023) showed good correlation to Eureka wind speed (R=0.58, p=0.002) for years that overlap, but not with Alert (R= -0.013, p=0.95) (Table S3, Fig. S8). Based on the stronger relationships between Parks Canada MAAT (Parks Canada with permission, temperature available between 1989-2023) and wind speed data at nearby Tanquary Fiord Airport and on Lake Hazen itself (Parks Canada with permission, temperature data available between 1988-2009, wind speed data available between 1988-2009) (Fig. S8), Eureka was chosen to best represent Lake Hazen (Table S3).

For GBL, the Délįnę weather station located on the shoreline of the lake on Keith Arm (Environment Canada station id 22010KA, temperature data from 1991-2007, no wind data) is ideally located but was too short for our purposes; however, this record was used to assess similarities in trends during overlapping years with nearby Norman Wells (MAAT 1943-2019, wind speed 1943-2017). The Tulita weather station (Environment Canada station id 2201700, temperature data from 1904-2017, no wind data) is located to the south of our coring locations on GBL and although it has the longest record, there were many gaps in the temperature data, particularly after 1920. The available years from the Tulita stations that overlapped with Norman Wells MAAT (1943-1957 and 1992-2007) were highly correlated (Table S3, Fig. S9), but the overall temperatures were higher at the southern Tulita station. Although the overall temperature trends were similar, the actual temperatures recorded at the Tulita station were not considered a good match for GBL and were not used to extend the record back in time. Norman Wells was chosen to represent GBL as this was the only nearby weather station with wind speed data, had the longest and most continuous record for MAAT, and temperature data were highly correlated to overlapping years (1991-2007) recorded at the Délįnę weather station (R = 0.79, p = 0.0007) (Table S3). The Hay River weather station was chosen to represent GSL as it has the longest, most complete air temperature and wind speed data in the region and is closer to the West Basin coring sites than the Yellowknife weather station. This station also has ground observations of lake ice until the 1990s. <u>Significant points of change in MAAT and wind speed data:</u> Piecewise linear regressions were applied to the MAAT and wind speed data for the three stations to identify significant points of change (breakpoints) after assessing the best-fit model (2 or 3 segments) for each climate metric at each station (based on R^2 and p-values). The mean of the significant breakpoint dates (across metrics and stations) was used in Fig. 3 (main paper) to represent the period of accelerated climate change (shaded area).

Table S3: **Comparisons among various available weather station data for GBL, GSL and Lake Hazen.**

**LAKE HAZEN**	**N**	**Correlation**	**P-value**	**Notes**
**MAAT**				
Eureka vs. Alert	68	0.85	<0.00001	
**Eureka vs. Tanquary**	29	0.40	**0.007**	
**Eureka vs. Hazen**	16	0.59	**0.017**	
**Alert vs. Tanquary**	27	0.38	**0.048**	
Alert vs. Hazen	16	0.28	0.29	
Tanquary vs. Hazen	14	0.48	0.83	Many gaps
**WIND SPEED**				
Eureka vs. Alert	60	0.22	0.09	Different
**Eureka vs. Tanquary**	18	0.61	**0.007**	6 gaps
Alert vs. Tanquary	16	0.09	0.74	8 gaps
Eureka vs. Hazen	*8*			
Alert vs. Hazen	*8*	*Too many missing data points to perform analyses*		
Tanquary vs. Hazen	*8*			
**GREAT BEAR LAKE**				
**MAAT**				
**Délı̨nę vs. Norman Wells (1991-2007)**	14	0.79	**0.0007**	3 gaps
**Tulita vs. Norman Wells (1943-1957)**	13	0.84	**0.0003**	1 gap
**Tulita vs. Norman Wells (1992-2007)**	14	0.63	**0.015**	2 gaps
**WIND SPEED**				
Norman Wells station only		na		
**GREAT SLAVE LAKE**				
**MAAT and WIND SPEED**				
Hay River (same as in (1) Rühland et al. 2023		na		

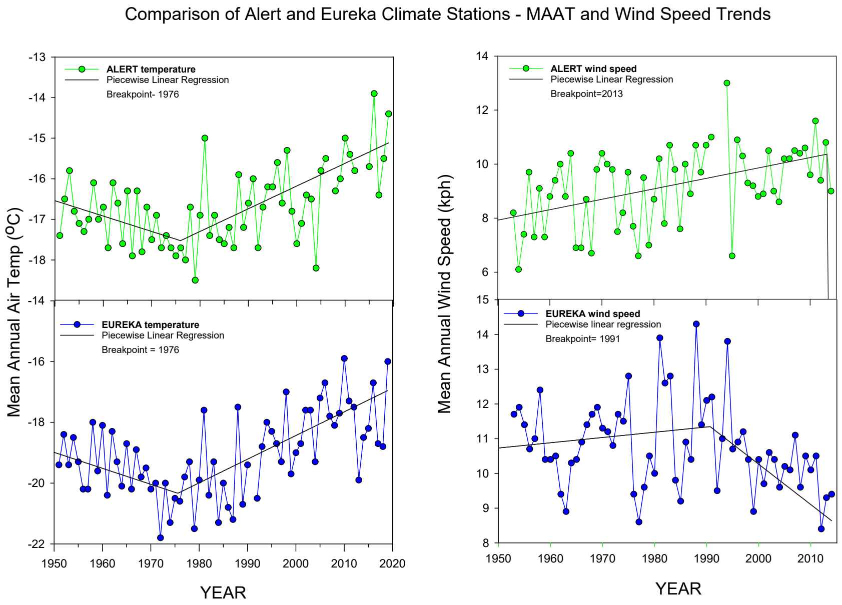

Fig. S7. Comparisons between Alert and Eureka weather station data (MAAT and wind speed).

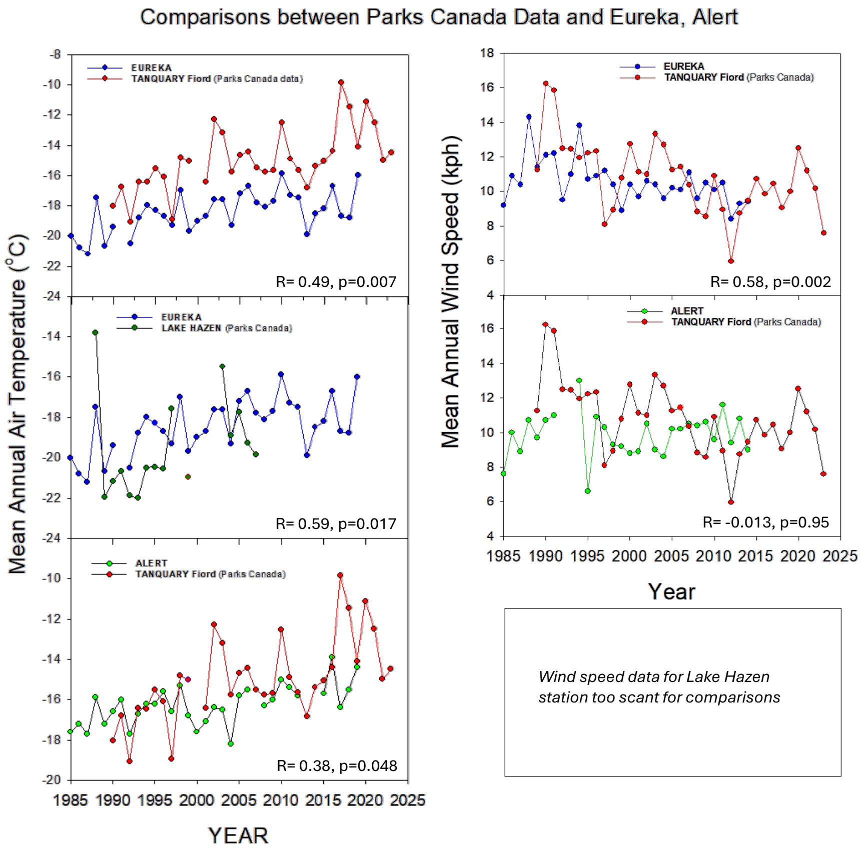

Fig. S8. Comparisons between Eureka weather station data and the Parks Canada data collected from Tanquary Fiord and Lake Hazen.

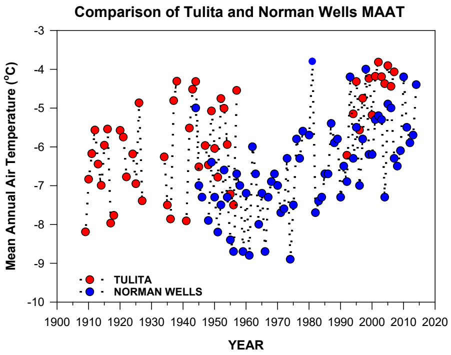

Fig. S9 Comparisons between MAAT from Norman Wells and Tulita climate stations.

Ice phenology records

Trends in the number of ice-free days for the three lake regions were based on existing ground observations (GSL. Hay River) available between 1956 and 1991, passive microwave remote sensing data between 1980 to 2017 (GBL and GSL; ref. 15), and from examining NASA daily satellite images available since 2000 (Lake Hazen, data provided by D. Muir). Ground observation ice records were short (GBL) or do not exist (Lake Hazen). The 54-year lake ice record for GSL (15) combines ground observation data (station id WRS48) accessed from the National Snow and Ice Data Center (NSIDC) (ref. 16; https://doi.org/10.7265/N5W66HP8) with remote sensing data between 1992 and 2017, provided to the authors by Su et al. (15). Likewise, remote sensing ice phenology data were also provided to the authors by Su et al. (15) for GBL.

Great Bear Lake: Ground observation ice records for the GBL region were generally short and did not exist beyond the 1990s, a period of interest for this study. Ground observation data that were available for the region included from the Mackenzie River (~1960 to 1992), Great Bear River (1956, 1958) and from GBL at Port Radium (1952-1973). To assess whether the longer Mackenzie River record is representative of GBL ice phenology trends, comparisons were made to remote sensing data by Su et al. (15) as well as data extracted by viewing daily images between 2000 and 2022 from NASA MODIS satellite data. The Mackenzie River ice data have poor relationships with Su et al. (15) data (where years overlap), indicative of differences between ice phenologies on the river and on the lake itself. The data were weakly correlated and not significant - the ice-free days were substantially higher overall in the river and therefore likely not the best representation of ice phenology changes on the lake (Table S4). This is not surprising given that warm water coming from the south into the Mackenzie River would result in an earlier ice break-up than in the lake.

Table S4. Comparisons of ice phenology trends from Mackenzie River ground observations, NASA MODIS daily satellite images, and remote sensing data from Su et al. (15) for Great Bear Lake (GBL).

GBL # ice-free days comparisons	Years	Correlation	P-value
Site **B** (Su et al. 2021) & McVicar Arm (NASA images)	2000-2017	**0.79**	0.001
Site **C** (Su et al. 2021) & McVicar Arm (NASA images)	2000-2017	**0.82**	0.00003
Site **B** (Su et al. 2021) & Smith Arm (NASA images)	2000-2017	**0.84**	0.00001
Site **C** (Su et al. 2021) & Smith Arm (NASA images)	2000-2017	**0.67**	0.002
Site **B** (Su et al. 2021) & Mackenzie R. (ground obs.)	1979-1994	0.34	0.22
Site **C** (Su et al. 2021) & Mackenzie R. (ground obs.)	1979-1994	0.28	0.31

The number of ice-free days estimated through remote sensing at sites B and C in the Su et al. (15) study (nearest to our coring sites- Fig. S10) were calculated from the number of days between the break-up end date (BUE) and the freeze-up end date (FUE). To assess whether these satellite data were a reasonable representation of ice phenology on GBL, these data were compared to data extracted from NASA MODIS satellite images where daily images were examined to determine trends in ice-off day of year and ice-on day of year at the tips of each arm (Smith, Keith and McVicar) (Fig. S10). Observations were focused on the tips of the arms because many of the satellite images for Keith Arm as well as images towards the middle of the lake were not available from November to February for most years (they were blacked out). Aside from that issue, most days were remarkably clear and therefore, we were able to determine the day that ice covered those parts of the lake and the day that ice left those parts of the lake for most years. Trends in the GBL remote sensing data were highly correlated to ice phenology data the authors extracted from observing NASA MODIS daily satellite images between 2000 to 2022 (Table S3). For Smith and McVicar arms, there were strong and significant correlations between the number of ice-free days determined from NASA satellite images data and the remote sensing data for Site B and Site C (2) for 17 overlapping years (2000 to 2017) – see Table S3. Keith Arm data only consisted of ice-off day of year and therefore, the number of ice-free days could not be calculated. This assessment provides confidence that the longer ice record (1978 – 2017) supplied by Su et al. (15) is a reasonable representative of GBL ice phenology trends (and by extension, remote sensing ice phenology used for GSL) for this study.

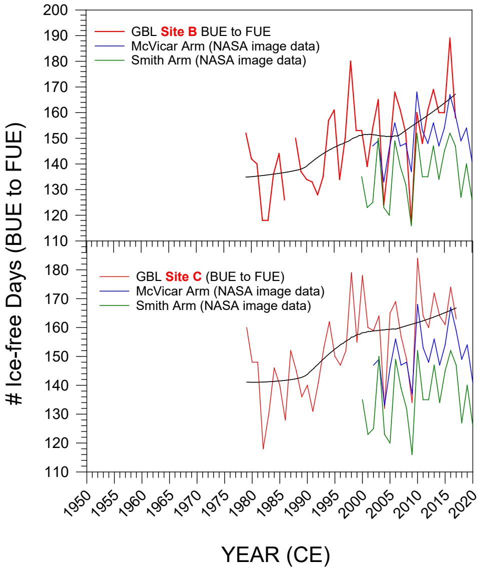

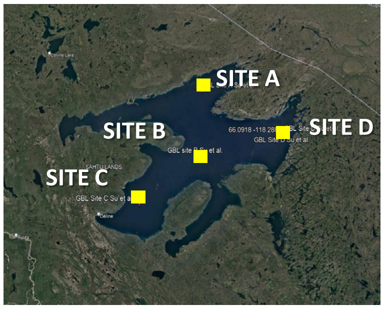

Fig. S10. Great Bear Lake (GBL) # of ice-free days. Comparisons between ice-free duration (# ice-free days) derived from NASA MODIS images and from remote sensing data from Su et al. (15). Map on right shows the locations of sites (A-D) on GBL referred to in Su et al. (15). BUE = Break-up End Date; FUE = Freeze-up End Date.

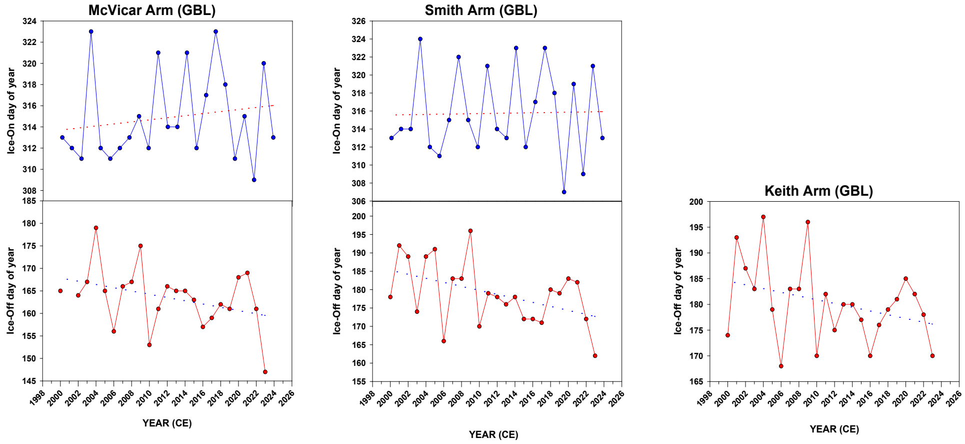

Fig. S11: Ice Phenology data for Great Bear Lake (GBL) extracted from examining daily satellite images (NASA MODIS) https://worldview.earthdata.nasa.gov/. The plots of ice-on and ice-off for GBL above nicely show ice phenology changes on GBL are largely driven by earlier ice-off dates. Ice-on dates are later (as would be expected with warming), but not as dramatic as the ice-off dates. These were used to calculate #ice-free days and compared to remote sensing data (15) to determine whether the latter were suitable for comparing to paleolimnological data.

Lake Hazen: Ice records available for the remote Lake Hazen are few and these include what was published in Lehnherr et al. (17) (from 2000 to 2012), with updates (2000 to 2022) by Hudelson et al. (18) using data derived from NASA MODIS satellite images of northern Ellesmere Island (break up, freeze-up, and ice-free dates). Although relatively short, the number of ice-free days shows a clear increasing trend (upper panel in Fig. S12).

GSL: Ice phenology data used in Rühland et al. (1) were also used in this study. The #ice-free days from 1960 – 1990 (Hay R. Ground observations) were combined with the # ice-free days (BUE to FUE: Break-up end date to Freeze-up end date) from Su et al. (15) remote sensing data. Note that ice phenology data using daily MODIS satellite images from NASA were attempted for GSL. However, there were a high number of very cloudy days during the periods of ice-on and ice-off on the lake in numerous years, and therefore this approach was abandoned. However, the strong agreement between Su et al. (15) data and the ice data derived from daily satellite images from NASA for GBL, by extension, provides confidence that the Su et al. (15) data are representative of the ice phenology for the West Basin. In addition, comparison between Su et al. (15) remote sensing data with the ground observation data at Hay River yielded strong relationships and therefore were added together to make a longer, composite record (1).

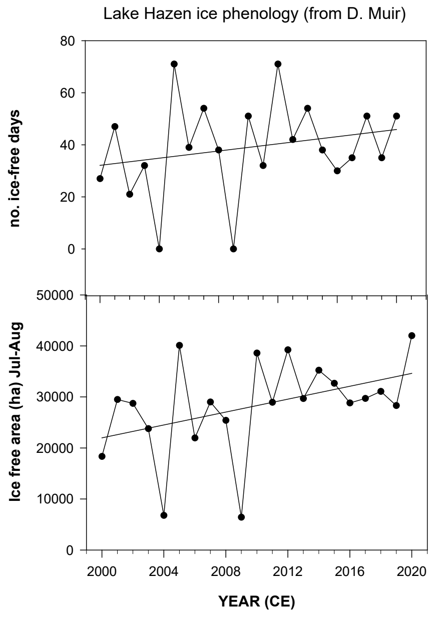

Fig. S12. **Ice phenology data for Lake Hazen** extracted from examining daily satellite images (NASA MODIS) https://worldview.earthdata.nasa.gov/. Ice-free area and ice-free days (2000 to 2020) derived from break-up, freeze-up dated – courtesy Derek Muir.

References

1. K.M. Rühland, M. Evans, J.P. Smol, Arctic warming drives striking twenty-first century ecosystem shifts in Great Slave Lake (Subarctic Canada), North America's deepest lake. Proc. R. Soc. B 290: 20231252 (2023).
2. N. Michelutti *et al*., Contrasting the ecological effects of decreasing ice cover versus accelerated glacial melt on High Arctic's largest lake. Proc. R. Soc. B **287**, 20201185 (2020).
3. Schelske et al. (1994). Schelske CL, Peplow A, Brenner M, Spencer CN. 1994 Low-background counting: applications for ^{210}Pb dating of sediments. *J. Paleolimnol.* **10**, 115-128.
4. Appleby PG. 2001 Chronostratigraphic techniques in recent sediments. *In*: Last WM, Smol JP (eds) Tracking environmental change using lake sediments, basin analysis, coring, and chronological techniques, vol 1. Springer, Dordrecht, pp 171–203
5. K.M. Rühland, J.P. Smol, Freshwater diatoms from the Canadian Arctic treeline and development of paleolimnological inference models. J. Phycol. 38: 249-264 (2002).
6. K. Krammer, H. Lange-Bertalot, Bacillariophyceae, parts 1–4. *In*: Subwasserflora von Mitteleuropa. Fischer Verlag, Stuttgart, Germany (1986–1991).
7. K.E. Camburn, D.F. Charles, Diatoms of Low Alkalinity Lakes in the Northeastern United States. Philadelphia: Academy of Natural Sciences of Philadelphia, Scientific Publications, 152 pp. (2000).
8. H. Tanaka, Taxonomic Studies of the Genera *Cyclotella* (Kützing) Brébisson, *Discostella* Houk et Klee and *Puncticulata* Håkansson in the Family Sephanodiscaceae Glezer et Makarova (Bacillariophyta) in Japan. Vol. 53 Stuttgart, J. Cramer. (2007).
9. H. Häkansson, H. Kling, A light and electron microscope study of previously described and new *Stephanodiscus* species (Bacillariophyceae) from central and northern Canadian lakes, with ecological notes on the species. *Diat. Res.* **4**, 269-288 (2011). https://doi.org/10.1080/0269249X.1989.9705076
10. E.D. Reavie, A.R. Kireta, Centric, Araphid and Eunotioid Diatoms of the Coastal Laurentian Great Lakes. *Bibliotheca Diatomologica* **62**,1-184 (2015).
11. N. Michelutti et al. Do spectrally inferred determinations of chlorophyll *a* reflect trends in lake trophic status? J. Paleolimnol. 43: 205-217 (2010).
12. N. Michelutti, J.P. Smol, Visible spectroscopy reliably tracks trends in paleo-production. J. Paleolimnol. 56: 253-265 (2016).
13. M. Sayers, K. Bosse, G. Fahnenstiel, R. Schuchman. Carbon fixation trends in eleven of the world's largest lakes: 2003–2018. Water **12**, 3500 (2020).
14. C.I. Jackson, The vertical profile of wind at Lake Hazen, N.W.T. Arctic 1**8**, 21-35 (1965). doi 10.14430/arctic3447 (14)
15. L. Su, T. Che, L. Dai, Variation in ice phenology of large lakes over the Northern Hemisphere based on passive microwave remote sensing data. Remote Sens. **13**, 1389 (2021). (15)
16. B. Benson, J. Magnuson, S. Sharma, Global lake and river ice phenology database, version 1. Boulder, CO: NSIDC, National Snow and Ice Data Center (2000). (16)
17. I. Lehnherr *et al*., The world's largest High Arctic lake responds rapidly to climate warming. Nat. Comm. **9**, 1290 (2018). (17)
18. K. Hudelson *et al*., Mercury at the top of the world: A 31-year record of mercury in Arctic char in the largest High Arctic lake, linked to atmospheric mercury concentrations and climate oscillations. Env. Pollut. **337**, 1224566 (2023). (18)
